# Therapeutic efficacy of artemether-lumefantrine plus single low dose primaquine for the treatment of uncomplicated *Plasmodium falciparum* malaria in a high transmission setting, Western Ethiopia

**DOI:** 10.1371/journal.pone.0335833

**Published:** 2026-07-17

**Authors:** Jimma Dinsa Deressa, Sinknesh Wolde Behaksra, Eshetu Molla, Alemayehu Letebo, Girma Shumie, Bethlehem Adnew, Dawit Hailu Alemayehu, Fikregabrail Aberra Kassa, Tamrayehu Seyoum, Elias B. Tafa, Kidist Woldekidan, Legesse Alamerie Ejigu, Tiffany Huwe, Migbaru Keffale, Gudissa Assefa Bayissa, Cristian Koepfli, Bayissa Chala, Yehenew Asmamaw, Fitsum Girma Tadesse, Endalamaw Gadisa

**Affiliations:** 1 Armauer Hansen Research Institute, Malaria and Neglected Tropical Diseases Research Division, Addis Ababa, Ethiopia; 2 Department of Applied Biology, School of Applied Natural Science, Adama Science and Technology University, Adama, Ethiopia; 3 Addis Ababa University, Addis Ababa, Ethiopia; 4 Department of Biological Science, University of Notre Dame, Notre Dame, United States of America; 5 Ministry of Health, Malaria elimination program, Addis Ababa, Ethiopia; George Washington University School of Medicine and Health Sciences, UNITED STATES OF AMERICA

## Abstract

**Background:**

The development and spread of drug-resistant parasites continue to threaten progress toward malaria elimination. Therapeutic efficacy and molecular resistance marker studies are needed to guide national control programs. In African settings, evidence of partial resistance to artemisinin-based combination therapies (ACTs) associated with *Pfkelch13* mutations is accumulating, and World Health Organization (WHO) recommends regular monitoring of first line antimalarial drugs for early detection of resistant parasites. In this study, we evaluated the efficacy of artemether-lumefantrine (AL) combined with a single low dose of primaquine (PQ) for treating uncomplicated *Plasmodium falciparum* malaria in a co-endemic area where *P. falciparum* predominates.

**Methods and findings:**

One hundred twenty-three patients with *P. falciparum* mono-infection were enrolled between November 2020 to March 2021 and treated with artemether-lumefantrine (AL) plus a single low dose of primaquine (PQ) as per the national malaria treatment guideline and followed up for 28 days. Ethical approval was obtained from the AHRI/ALERT ethics committee (Po/23/19), and the study was registered at Pan-African clinical trials registry (PACTR) with unique identification number of PACTR202509595696440. *Pfmsp2* capillary electrophoresis (CE) genotyping was used to differentiate recrudescence from new infections. More than half (56.1%) of the participants had high parasitemia (>10,000 parasites/μL) at enrollment. On day 3, 16.9% (20/118) remained parasitemic, and of the 10 individuals with detectable gametocytes at enrollment, only 3.4% remained gametocytemic on day 3, and 100% parasite clearance was observed on day 7, respectively. Multiplicity of infection was 3.8 at enrollment and 1.7 at the time of recurrence. The adequate clinical and parasitological responses at 28-day (ACPR) of per protocol analysis (PPA) was 73.7% for PCR-uncorrected and 91.3% for PCR-corrected, respectively and while, the intention-to-treat analysis (ITA), the Kaplan–Meier estimated treatment success at day 28 was 93.2% (95% CI: 88.5–98.2) after PCR correction, compared with 78.3% (95% CI: 71.0–86.4) in the PCR-uncorrected analysis. In our study assessment, no cases of severe malaria or serious adverse events occurred.

**Conclusions:**

The efficacy observed in this study, although remaining above the WHO policy change threshold after PCR correction, may indicate a potential decline in AL’s effectiveness in this high transmission setting. However, because antimalarial drug concentrations were not measured and evening doses were not fully directly observed, reduced drug exposure or imperfect adherence cannot be excluded as possible contributors to the observed treatment outcomes Therefore, we suggest regular therapeutic efficacy monitoring and further investigation using advanced molecular techniques, such as next-generation sequencing (NGS), to enable early detection of resistance-associated parasite variants that may compromise treatment efficacy.

## Introduction

Malaria remains an important public health concern globally [1,2]. As per the World Health Organization (WHO) estimate, there were 282 million malaria cases and 610,000 associated deaths registered globally in 2024, according to the most recent World Malaria Report, marking a slight increase from 2023 [3,4]. Between 2000 and 2015, the wide implementation of integrated control interventions resulted in an 18% and 48% reduction in malaria incidence and mortality, respectively [5]. During the same period, 663 million malaria cases were averted in sub-Saharan Africa, of which 21% were due to ACTs [5]. Malaria is a major health and socioeconomic challenge with over 81 million Ethiopians living at risk of malaria according to the national malaria strategic plan 2024/25–2026/27 (Draft NMSP, MOH). *P. falciparum* malaria remained the major cause of morbidity and mortality. Artemether-lumefantrine (AL) is the current first-line treatment for uncomplicated *P. falciparum* in Ethiopia. In line with the national malaria elimination strategy, a single low-dose of primaquine (0.25 mg/kg) is administered alongside AL to reduce gametocyte carriage and interrupt transmission [1].

Ethiopia’s over five decades of malaria control program achieved a substantial decrease in related mortality and morbidity until 2018. Yet, the gains were eroded, and the country witnessed a surge in cases through 2019−23 (S1 Fig). The advent of the COVID-19 pandemic, *hrp2*/*3* gene deletions, internal conflicts, and invasion of *An. stephensi* were some of the attributed causes [6].

The detection of gene variants implicated in the therapeutic efficacy of artemisinin combination therapy (ACT)-based treatment called for increased vigilance in African [7]. Studies carried out in Rwanda and Uganda revealed mutations in the *kelch13* region, *R561H,* and *A675V,* linked to delayed parasite clearance [8,9]. Recrudescence following AL treatment linked to wild-type *Pfcrt K76T* and *Pfmdr1 N86Y* gene variants were reported in Kenya, Tanzania, and Zanzibar [10–13]. The K13 mutation implicated in AL partial resistance in Ethiopia, Sudan, and Eritrea (S2 Fig),  showed an increased detection rate over time [14,15]. In a *P. falciparum* and *P. vivax* co-endemic setting, in samples from AL plus a single low-dose PQ therapeutic efficacy trial, we genotyped the samples via capillary gel electrophoresis by targeting allelic variants of *msp2* of FC27 and 3D7 to quantify multiplicity (complexity) of infection and differentiate the recrudescence from new infection.

## Methods

### Study area and period

**Fig 1:**
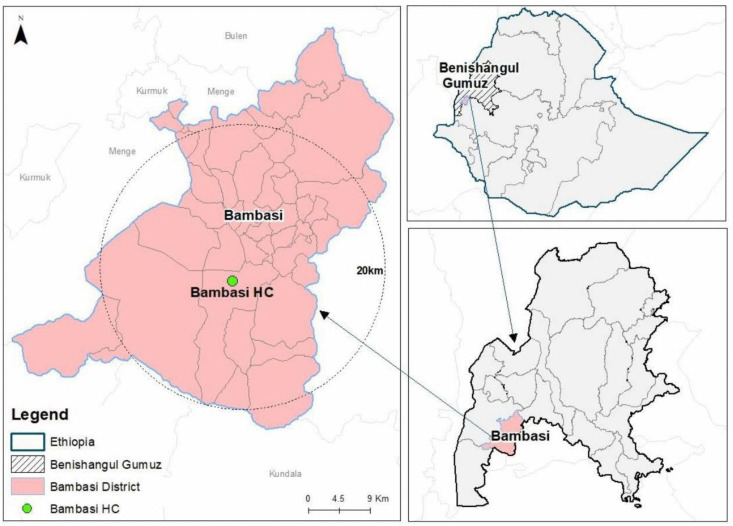
Map of the study site, Bambasi health center, Bambasi district, Benishangul Gumuz regional state, Northwest Ethiopia. Administrative data were obtained from geoBoundaries dataset (William & Mary, CC BY 4.0).

This study was conducted at Bambasi health center (9°45′N 34°44′E), Bambasi district (Fig 1) from November 2020 to March 2021. The study map was created by QGIS software 3.4 with open licensed data sourced from Ethiopian administrative geoBoundaries dataset (geoBoundaries) to ensure full compliance of copyright with CC BY 4.0 license [16]. *P. falciparum* was the dominant parasite species in the study setting [17]. The peak transmission season in the area is from September to December, and *An. gambiae sensu stricto* (and presumably *An. arabiensis*) is the primary vector [18].

### Study design, sampling and recruitment

A prospective single arm partially observed study was done following the methods for surveillance of antimalarial drug efficacy by WHO [19] and additionally, this in vivo efficacy trial study was registered under Pan-African clinical trials registry (PACTR) with unique identification number of PACTR202509595696440. The sample size was calculated according to the WHO protocol for surveillance of antimalarial drug efficacy studies: n = [Z^2^ × p(1 − p)]/d^2^. Assuming a 95% confidence level (Z = 1.96), an expected artemether-lumefantrine treatment failure rate of 5% (p = 0.05), and a precision of 5% (d = 0.05), the minimum sample size of 88 was required. To account for the high malaria transmission intensity in Western Ethiopia, which increases the likelihood of re-infections and loss to follow-up, the sample size was inflated by approximately 40%. Consequently, 123 participants were enrolled to ensure adequate power for the 28-day PCR-corrected efficacy analysis.

Self-reporting febrile patients with axillary temperature ≥ 37.5˚C or a history of fever within the previous 24 hours at the time of diagnosis, and who fulfilled the study inclusion criteria, were screened for enrollment. Microscopy and RDT confirmed uncomplicated *P. falciparum* mono-infected, 6 months or older, body weights >5 kg, hemoglobin level 5.0 g/dL and above, parasitemia ≥500 parasites/μL. Ethiopia is globally classified as a low-transmission setting [20]. The Ethiopian Ministry of Health defines transmission based on the annual parasite index (API): 0 (malaria-free), < 10 (low), 10–50 (moderate), and >50 (high) [21]. Even though Bambasi is classified as high transmission setting in local context, as per the WHO stratification, it falls under low transmission setting. Prior to the COVID-19 pandemic, extensive vector control interventions and artemisinin-based combination therapies significantly reduced local transmission intensity and patient volume (S1 Fig). Consequently, the national malaria control program approved our study protocol with a lowered threshold of ≥500 parasites/μL to monitor AL + single low dose PQ efficacy at the Bambasi site. Lowering this limit is essential to ensure adequate participant enrollment in areas with declining transmission intensity. Furthermore, WHO guidelines note that partial immunity from repeated exposure can lead to spontaneous parasite clearance and low-grade parasitemia. Given the site-specific reduction in transmission and seasonal variability, the ≥ 500 parasites/μL threshold was necessary to capture the at-risk population. This ensured adequate clinical representation to evaluate intervention effectiveness in a real-world setting where asymptomatic and low-parasitemia cases predominate.

In addition, per protocol, inclusion criteria included a negative pregnancy test (HCG) for women of childbearing age, non-breastfeeding status, and residence within a 20 km radius of Bambasi Health Center to facilitate follow-up. In addition, as per the study protocol, a negative pregnancy test (HCG) for women of childbearing age, non-breastfeeding status, and residence within a 20 km radius of Bambasi health center were included to facilitate follow-up. Written informed consent was obtained from all adult participants and from the parents or legal guardians of participating children. For participants aged 11–17 years, additional written assent was secured. During enrolment and all subsequent follow-up visits, body temperature was measured using digital axillary thermometers. Clinical and sociodemographic data were collected by trained clinical nurses and public health officers, then stored in a secure REDCap (Research Electronic Data Capture) database accessible only to authorized study investigators.

### Drug regimens and follow-up

The study followed the national guideline to treat cases using the standard first-line regimen of artemether-lumefantrine (AL) plus single low-dose primaquine [22]. Artemether-lumefantrine (AL, 20 mg of artemether and 120 mg of lumefantrine; Novartis Pharmaceutical Corporation, New York, NY, USA) was administered twice daily for three consecutive days. The morning AL doses were administered under direct observation at the health facility. The evening doses were taken at home due to logistical constraints related to geographic accessibility and to minimize participant burden in this rural setting. Adherence to these home-administered evening doses was monitored during the subsequent morning visit through participant interviews and inspection of the returned medication blister packs for pill counts. A single dose of primaquine (PQ) (0.25 mg/kg) was given on the day of enrollment, 30 minutes after the first dose of AL. To enhance lumefantrine absorption, participants were given vanilla cream biscuits (6*125g) with 0.5 L bottled water during drug administration and advised taking the evening doses after a meal. Participants were observed for 30 minutes after drug administration, and if vomiting occurred within this period, the full dose was re-administered. Participants received an appointment card indicating scheduled follow-up visits on days 1, 2, 3, 7, 14, 21, and 28 after dosing or at any unscheduled time when they had any of the malaria symptoms and/or hemolysis (darkening of their urine). The appointment card included the visit dates and pictorial instructions illustrating symptoms of hemolysis. Further, they were communicated by phone a day earlier to the scheduled date; those who did not show up on schedule were visited at their residence for appropriate drug administration.

### Withdrawal and loss to follow-up

Participants were excluded from the study if they experienced a second vomiting during the first dose of AL, took self-prescribed antimalarial drugs, missed doses of AL, or were diagnosed with other than falciparum. Moreover, participants who could not attend the health center on the scheduled date and could not be reached by the research team/tracer within 1 or 2 days after the scheduled visit date were considered as a loss to follow-up. Although the study protocol allowed for the enrolment of both species, this analysis focuses exclusively on *P. falciparum*. Consequently, individuals with *P. vivax* mono-infections or mixed infections identified at enrolment were excluded from this study. Furthermore, any participant who developed *P. vivax* or mixed infection during the 28-day follow-up period was withdrawn from the efficacy analysis and treated with radical cure therapy according to national guidelines [22]. These cases were treated as censored observations in the Kaplan-Meier analysis at the time of detection.

### Laboratory procedures

Blood samples were collected from finger-prick for multi species CareStart rapid diagnostic test (RDT) (American Access Bio International Company) [23], thick and thin blood smears, hemoglobin assessment, and dried blood spot (DBS) for molecular analysis on days 0, 1, 2, 3, 7, 14, 21, and 28, and any unscheduled visit(s).

The thin smear was stained with 3% Giemsa and fixed in absolute methanol for 30–45 minutes and used for parasite speciation and quantification. Asexual parasite density per microliter was determined based on the number of parasites counted per 200 WBCs on a thick blood film. Two microscopists read the slides, and results with discordant by ≥400 parasites/µL were re-read by a third microscopist, blinded to the two results, and parasite density was calculated by averaging the two closest counts. Hemoglobin concentration was measured using a portable spectrophotometer (HemoCue®, Angelholm, Sweden), on days 0, 3, 14, and 28.

Genomic DNA was isolated from DBS using the Chelex method as described elsewhere [24,25]. The amplification of *msp2* targeting FC27 and 3D7 allele loci was conducted on paired samples. The purified PCR products were genotyped following the protocol (Applied Biosystems, Foster City, CA, USA) and analyzed using capillary electrophoresis (Macrogen, Inc., Korea) [26]. Then, the capillary electrophoresis (CE) sequence analysis was performed using GeneMarker®V2.7.0 software (State College, PA) for allele sizing and scoring by both trained experts of the University of Notre Dame and Armauer Hansen Research Institute (AHRI).

### Treatment outcomes

The primary outcomes of this study were to determine the clinical and parasitological response of patients to antimalarial treatment with the proportion of patients who achieve the complete parasite clearance and resolution of symptoms. The treatment outcomes were classified according to the WHO guidelines for surveillance of antimalarial drug efficacy. Late Clinical Failure (LCF) was defined as the presence of parasitemia between Days 4 and 28 accompanied by fever (axillary temperature ≥37.5 °C) or danger signs of severe malaria. Late Parasitological Failure (LPF), and Adequate Clinical and Parasitological Response (ACPR) were also defined according to the same WHO criteria [27,28]. The *Pfmsp2* genotyping was conducted to distinguish between new infections and recrudescence. The *msp2* amplicons were resolved through capillary electrophoresis (CE), allelic variants were identified and 3 base pairs or more differences were considered as new infections. Recurrent parasites are potentially classified into four based on the degree of allelic matching as earlier described in the study conducted by Mugittu et al 2006. These are; i) all alleles are identical at both baseline and recurrent parasite, ii) some alleles are missing in the recurrent parasites, iii) recurrent parasites contain alleles identical to those at baseline with additional/new ones not observed at baseline, and iv) all alleles in the baseline and recurrent parasites samples are different. It is generally accepted that categories from i-iii are represented as recrudescent while step four (iv) represents new infections [29].

### Data analysis

Data were entered into Microsoft Excel and imported into R-4.5.1version (Integrated Development for R Studio, PBC, Boston, USA) for statistical analysis. Descriptive statistics were used to calculate the frequency of MOI based on *Pfmsp2* capillary gel electrophoresis genotyping. Kruskal-Wallis’s/Fisher’s Exact Test, were used to measure statistical significance (P < 0.05) for specific outcomes, including differences in parasite clearance rates across age groups on follow-up days, and changes in anemia status between enrollment and follow-up days. Kaplan-Meier survival analysis was used to estimate the PCR-corrected and uncorrected ACPR on day 28.

## Results

### Participant characteristics

The mean age of participants was 17.8 years (range 3–62), with the majority being male (61.0%, n = 75), and most (57.7%) were in the 6–15 years age group. The geometric mean (GM) parasitemia at enrollment was 13,513/μL (Table 1). Complete concordance (100%) was observed between RDT and microscopy results, as both methods were required for study inclusion. All 123 participants tested positive for *P. falciparum* by both diagnostic tools at enrolment.

**Table 1:** Baseline characteristics of study participants stratified by age and sex.

Parameter	0.5-5y	6-15y	>=16y	Total	P-value
Sex, male, n (%)	3 (50%)	44 (62%)	28 (60.9%)	75 (61%)	0.863
Weight (kg), mean (SD)	15.2 (1.9)	33.8 (11.6)	54 (8.8)	40.4 (15.3)	< 0.001
Temperature (°C), Mean (SD)	37.6 (0.7)	37.4 (1)	37.5 (1.4)	37.5 (1.2)	0.85
haemoglobin (g/dL), mean (SD)	11.5 (2.4)	12.8 (1.8)	13.2 (1.7)	12.9 (1.8)	0.173
Parasite density (/uL), GM (95% CI)	10176.7 (1819.2-56927.8)	12797.3 (9881.9-16572.7)	15250.8 (9684.4-24016.8)	13513 (10730.5-17017.1)	0.74

Note: SD = Standard Deviation; GM = Geometric Mean; 95% CI = 95% Confidence Interval.

P-values were calculated using the Kruskal-Wallis’s rank-sum test for continuous variables (Weight, Temperature, Haemoglobin, Parasite Density) and Fisher’s Exact test for categorical variables (Sex) to compare baseline characteristics across age groups.

### Asexual blood stage parasite, gametocyte, and fever clearance rate

Parasite clearance rates were 51.7% (61/118) and 83.1% (98/118) on day 2 and day 3, respectively. The decline in parasitemia was more pronounced (91.3%, 42/46) in the 16 years and above age groups on day 1. Of the 10 participants with microscopically detected gametocytes at enrollment, 4 remained gametocytemic on days 3 and cleared all on day 7, respectively. Most individuals (99.2%, 119/120) became afebrile 48 hours after dosing, and complete fever clearance was attained by day 3 (Table 2). Although 16.9% (20/118) of participants remained microscopically parasitemic on Day 3, individual clearance trajectories illustrated a rapid log-reduction in asexual parasite density from baseline across the entire cohort (S3 Fig). No participants exhibited minimal decline or increasing parasitemia during the first 72 hours, confirming that the regimen was effective in the initial knockdown of the high baseline parasite loads observed in this setting.

**Table 2:** Parasitemia, gametocytaemia, and fever clearance rates by age group following treatment with AL plus single low-dose PQ.

Parameter	0.5-5y	6-15y	>=16y	Total	Statistic	P-value
Asexual parasitemia						
Day 0	6 (100%)	71 (100%)	46 (100%)	123 (100%)	–	–
Day 1	5 (83.3%)	62 (88.6%)	42 (95.5%)	109 (90.8%)	Fisher	0.266
Day 2	2 (40%)	42 (60.9%)	17 (38.6%)	61 (51.7%)	Fisher	0.064
Day 3	1 (20%)	15 (21.4%)	4 (9.1%)	20 (16.8%)	Fisher	0.172
Day 7	0 (0%)	0 (0%)	0 (0%)	0 (0%)	–	–
Day 14	0 (0%)	1 (1.5%)	0 (0%)	1 (0.9%)	Fisher	1
Day 21	1 (25%)	12 (18.8%)	4 (9.8%)	17 (15.6%)	Fisher	0.312
Day 28	1 (33.3%)	11 (22.4%)	6 (15.8%)	18 (20%)	Fisher	0.484
Fever (>37.5c)						
Day 0	4 (66.7%)	32 (45.1%)	22 (47.8%)	58 (47.2%)	Fisher	0.659
Day 1	1 (16.7%)	9 (12.9%)	7 (15.6%)	17 (14%)	Fisher	0.821
Day 2	0 (0%)	1 (1.4%)	0 (0%)	1 (0.8%)	Fisher	1
Day 3	0 (0%)	1 (1.4%)	0 (0%)	1 (0.8%)	Fisher	1
Day 7	0 (0%)	0 (0%)	0 (0%)	0 (0%)	–	–
Day 14	0 (0%)	0 (0%)	0 (0%)	0 (0%)	–	–
Day 21	0 (0%)	0 (0%)	0 (0%)	0 (0%)	–	–
Day 28	1 (33.3%)	2 (4%)	1 (2.6%)	4 (4.4%)	Fisher	0.158
Gametocytaemia						
Day 0	2 (33.3%)	4 (5.6%)	4 (8.7%)	10 (8.1%)	Fisher	0.066
Day 1	2 (33.3%)	6 (8.6%)	1 (2.3%)	9 (7.5%)	Fisher	0.037
Day 2	1 (20%)	4 (5.8%)	1 (2.3%)	6 (5.1%)	Fisher	0.203
Day 3	0 (0%)	4 (5.7%)	0 (0%)	4 (3.4%)	Fisher	0.285
Day 7	0 (0%)	0 (0%)	0 (0%)	0 (0%)	–	–
Day 14	0 (0%)	0 (0%)	0 (0%)	0 (0%)	–	–
Day 21	0 (0%)	0 (0%)	0 (0%)	0 (0%)	–	–
Day 28	0 (0%)	0 (0%)	0 (0%)	0 (0%)	–	–

*Note:* D = Day of follow-up. Values represent *n* (%). P-values indicate the statistical significance of differences in clearance rates across the three age groups, calculated using Fisher’s Exact test due to small cell counts (<5) in specific categories.

The proportion of anemia, as defined by the WHO Hb cut-off criteria [30], was 30% (37/123) on enrolment which declined to 20% at the end of the study (Table 3). There was no serious adverse event, the common adverse events reported were headache (35%), fever (21.8%), chills (14.7%), weakness (9.8%), nausea (6.5%), and abdominal pain (9.5%), respectively.

**Table 3:** Anaemia status by age group on each follow-up day, Bambasi Health Center.

Time Point	Status	0.5-5y	6-15y	>=16y	Total	P-value
Day 0	Anaemic	1 (16.7%)	11 (15.5%)	5 (10.9%)	17 (13.8%)	0.668
	Non-Anaemic	5 (83.3%)	60 (84.5%)	41 (89.1%)	106 (86.2%)	
	Total Analysed	6	71	46	123	
Day 3	Anaemic	2 (40%)	13 (18.8%)	8 (18.2%)	23 (19.5%)	0.459
	Non-Anaemic	3 (60%)	56 (81.2%)	36 (81.8%)	95 (80.5%)	
	Total Analysed	5	69	44	118	
Day 14	Anaemic	2 (40%)	6 (9%)	4 (9.3%)	12 (10.4%)	0.139
	Non- Anaemic	3 (60%)	61 (91%)	39 (90.7%)	103 (89.6%)	
	Total Analysed	5	67	43	115	
Day 28	Anaemic	1 (33.3%)	2 (4.1%)	2 (5.3%)	5 (5.6%)	0.203
	Non-Anaemic	2 (66.7%)	47 (95.9%)	36 (94.7%)	85 (94.4%)	
	Total Analysed	3	49	38	90	

***Note****:* Anaemia was defined as Haemoglobin < 11.0 g/dL (adjusted for age/altitude if applicable per national guidelines). Values represent *n* (%) of participants with valid haemoglobin measurements at each time point. *P-values* indicate the statistical significance of differences in anaemia prevalence across the three age groups, calculated using Fisher’s Exact Test. Missing data were excluded from the denominator of each time point.

### Treatment outcomes

**Fig 2:**
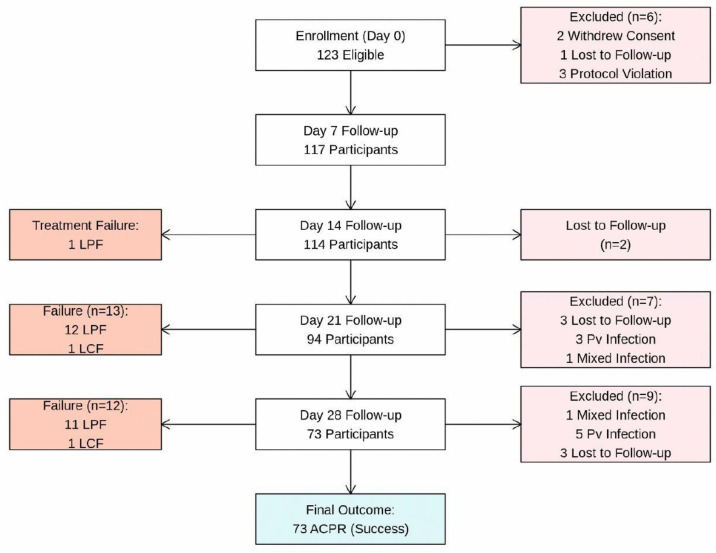
Study profile and participant flow: Schematic representation of enrollment, follow-up, and treatment outcomes. Participants classified as Late Parasitological Failure (LPF) or Late Clinical Failure (LCF) (left column) were included in the primary efficacy analysis as treatment failures. Participants who withdrew consent, were lost to follow-up, or were diagnosed with non-falciparum/mixed infections (right column) were excluded from the per-protocol analysis. Exclusions occurring between enrollment and Day 7 are detailed.

Adherence to the partially supervised treatment regimen was high; based on pill counts (blister counts) and participant interviews, *118/118 (100%)* of participants completed the full six-dose regimen as prescribed. No early treatment failures (ETF) were observed. Of the 123 enrolled participants, microscopically 73 achieved Adequate Clinical and Parasitological Response (ACPR) by Day 28. Twenty-six participants experienced treatment failure (LCF or LPF) and were included in the final efficacy analysis. 24 participants were excluded from the per-protocol analysis due to withdrawal of consent (n = 2), loss to follow-up (n = 12), withdrew due to protocol violation (n = 3), or detection of non-falciparum or mixed infections (n = 10*)* (Fig 2).

**Fig 3:**
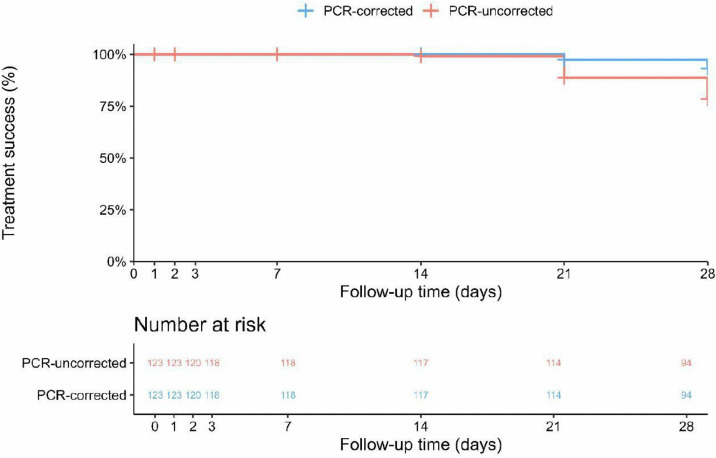
Kaplan–Meier estimates of treatment success during 28-day follow-up (PCR-corrected and PCR-uncorrected). Kaplan–Meier curves showing the cumulative probability of treatment success over 28 days of follow-up for PCR-corrected (blue) and PCR-uncorrected (red) analyses in the intention-to-treat population (n = 123). Treatment success represents the probability of remaining free of treatment failure during follow-up. In the PCR-corrected analysis, recurrent infections classified as reinfections by CE-genotyping were censored, whereas in the PCR-uncorrected analysis all recurrent parasitemia were considered treatment failures. Tick marks indicate censored observations. Numbers at risk at each follow-up day are shown below the plot.

The adequate clinical and parasitological responses at 28-day (ACPR) of per protocol analysis (PPA) was 73.7% PCR-uncorrected and 91.3% PCR-corrected, respectively (Table 4) and while, the intention-to-treat analysis (ITA), the Kaplan–Meier estimated treatment success at day 28 was 93.2% (95% CI: 88.5–98.2) after PCR correction, compared with 78.3% (95% CI: 71.0–86.4) in the PCR-uncorrected analysis (Fig 3).

**Table 4:** Efficacy of AL plus single low-dose PQ for the treatment of uncomplicated *falciparum* malaria, Bambasi health center, Bambasi district, Northwest Ethiopia, November 2020 to March 2021.

	Microscopy (CE-uncorrected)				msp2 (CE-corrected)			
Outcome	0.5 - 5	6-15	≥16	Total (n, %)	0.5 −5	6-15	≥16	Total (n, %)
ETF	0 (0)	0 (0.0)	0 (0.0)	0 (0.0)	0 (0)	0 (0.0)	0 (0.0)	0 (0.0)
LCF	0 (0)	3 (5.6)	0 (0.0)	3 (3.0)	0 (0)	2 (5.0)	0 (0.0)	2 (2.5)
LPF	0 (0)	16 (29.6)	7 (15.6)	23 (23.2)	0 (0)	3 (7.5)	2 (5.0)	5 (6.2)
ACPR	0 (0)	35 (64.8)	38 (84.4)	73 (73.7)	0 (0)	35 (87.5)	38 (95.0)	73 (91.3)
P-value*	–	–	–	0.038	–	–	–	0.432
Analyzed	0	54	45	99	0	40	40	80
Withdrawal	0	3	1	4	0	17	6	23
LFU	1	5	4	10	1	5	4	10
Protocol violation	0	6	4	10	0	6	4	10
Total	1	68	54	123	1	68	54	123

Note: ETF = Early Treatment Failure; LCF = Late Clinical Failure; LPF = Late Parasitological Failure; ACPR = Adequate Clinical and Parasitological Response; FU = Loss to Follow-up.

Analyzed: The per-protocol population excluding withdrawals and lost to follow-up.

Withdrawal: Includes protocol violations and, for the CE-corrected analysis, recurrences confirmed as new infections or undetermined by genotyping.

P-value*: Represents the statistical comparison of the therapeutic success rate (ACPR) across age groups using Fisher’s Exact test.

The Kaplan-Meier survival analysis (Fig 3) shows the cumulative proportion of success over 28 days. When stratified by age group, there was no statistically significant difference in survival times (Log-Rank Test, *p* = 0.3). Twenty-six recurrences were observed (uncorrected). On Day 28, four participants presented with fever (4.4%); however, only two participants met the WHO criteria for Late Clinical Failure (LCF) as they exhibited both fever and microscopically confirmed *P. falciparum* parasitemia. The remaining 24 recurrences were classified as Late Parasitological Failure (LPF). No cases of severe malaria or serious adverse events occurred. *Pfmsp2* CE genotyping was successfully amplified in 26 paired samples, identifying 98 allelic variants at day 0 (52 FC27, 46 3D7) and 44 at recurrence (29 3D7, 15 FC27). The mean MOI was 3.8 at enrollment and 1.7 at recurrence. Seven true treatment failures (5 LPF, 2 LCF) were confirmed as recrudescence by CE genotyping. The remaining 19 recurrences were classified as new infections or undetermined and were censored in the final analysis. Consequently, the PCR-corrected and uncorrected therapeutic efficacy as per ITA (Fig 3), the Kaplan Meier survival analysis demonstrated a significant divergence between the uncorrected and corrected curves, highlighting the high rate of re-infection in the study area. There was no statistically significant difference in therapeutic efficacy across age groups (Fisher’s Exact Test, *p* = 0.269).

## Discussion

Antimalarials remained at the top of the list of armaments in the malaria control and elimination endeavor. As malaria control interventions intensify, the selective pressure on parasite populations increases, leading to the emergence and spread of resistant strains that can evade current diagnostic and therapeutic tools. *Plasmodium falciparum* (*P. falciparum*) developed resistance to almost all antimalarials rolled out, thus surveillance strategies to continuously monitor such development have been put in place [20,31]. The combination of *in vivo* therapeutic efficacy study and CE based molecular genotyping was employed in identifying recrudescence, is used in these efficacy monitoring studies. Here we present a study in which we evaluated AL plus single low-dose PQ for the treatment of uncomplicated *P. falciparum* malaria, implicated to assess the efficacy of the combination therapy.

Our study site is a *P. vivax* and *P. falciparum* co-endemic area where *P. falciparum* is the predominant [17] with a high multiplicity of infection; we found 98 *msp2* allelic variants and an MOI of 3.8 at recruitment and 1.7 on the day of recurrence, respectively. We suggest that the observed decline in MOI post-treatment likely due to a combination of selective pressure exerted by the treatment, possibly leading to the survival of more resistant genotypes, and a reduction in genetic diversity as immune response and treatment clears susceptible strains. In areas with a high MOI, the WHO recommends amplicon-based sequencing [32] for identifying recrudescence, and combinations of *msp1*/*msp2*/*glurp* genotyping were used to distinguish new infections from recrudescence [33]. We used *msp2* capillary gel electrophoresis at a 3-base pair (bp) difference for the two allelic variants (3D7-FC27), and thus, efficacy results should be interpreted with caution.

Our findings indicated a 28-day PCR-corrected efficacy of 91.3%, which is lower than previously reported efficacy rates for AL in Ethiopia and this suggests the potential challenges for malaria control program. Efficacy rates can vary significantly by regional variability due to local transmission intensity, parasite strains, nutritional status, co-morbidity, treatment adherence, and severity of malaria during enrollment. Higher genetic diversity of the parasite can influence and lead to variations in susceptibility to AL, potentially, resulting lowering in overall efficacy as observed in our study conducted in high transmission setting with observed high complexity of infections. However, a systematic review and meta-analysis of 15 therapeutic efficacy studies that involved 1523 patients conducted from 2004 to 2020 documented a PCR-adjusted pooled success of 98.7% [2]. A more recent study done in two sentinel sites, western and southern Ethiopia, reported 100% treatment success after PCR adjustment [34]. A study done in Benishangul-Gumuz in 2020, close to our study site, reported 96% AL efficacy after PCR adjustment [35]. In our study, 88.3%, 48.3%, and 16.9% of participants had microscopy detectable parasitemia on day 1, 2 and day 3, respectively, while 100% clearance was achieved by day 7; residual submicroscopic and microscopic parasitemia raise concerns about treatment outcomes.

In the aforementioned study in Benishangul Gumuz [35], 38% and 10% of the participants remained microscopy positive on day 1 and day 2. Interestingly, 60% and 28% of participants had PCR detectable infection on days 3 and 7, respectively, while 20% of participants remained PCR positive at the last visit (day 28). In our study, AL + PQ reduced gametocyte carriage, with complete clearance by day 7, but prolonged clearance in 3.4% individuals (4 participant gametocytemic on day 3 suggests that while the WHO-recommended single low-dose primaquine (0.25 mg/kg) accelerates gametocyte clearance, it may not achieve immediate sterilization or complete clearance in all patients by Day 3. However, since we did not assess gametocyte viability or infectivity, the potential transmission-blocking effect cannot be ruled out despite the microscopic presence of gametocytes. This aligns with studies indicating that single low-dose PQ enhances gametocyte clearance but may not fully sterilize gametocytes [38]. Even though the smallest number of patients with detectable gametocyte observed during enrollment, the combination of AL and PQ demonstrated improved gametocyte clearance compared to AL alone, although the duration of gametocyte clearance was prolonged, with 4 participants remaining gametocytemic at day 3. Baseline factors, including pretreatment parasite density and hemoglobin levels, are known to influence clearance dynamics. However, our findings suggest that the addition of single dose primaquine significantly accelerates gametocyte clearance compared to artemether-lumefantrine (AL) monotherapy. Evidence from multiple high-transmission settings in Ethiopia supports this conclusion. A recent randomized controlled trial [36] and therapeutic efficacy studies in Arba Minch [37] and Setit Humera [38] consistently reported that with AL alone, complete gametocyte clearance was delayed until Day 14. In contrast, our cohort treated with AL plus SD-PQ achieved 100% clearance by Day 7, demonstrating a marked reduction in the duration of potential infectivity consistent with the enhanced sterilization effect of primaquine. It is important to note that gametocyte viability and infectivity were not directly assessed in this study. While microscopy confirmed the physical clearance of gametocytes, it cannot distinguish between viable parasites and those that may have been sterilized by primaquine prior to clearance [39]. Furthermore, as gametocyte viability and infectivity to mosquitoes were not assessed via membrane feeding assays, the presence of circulating gametocytes detected by microscopy post-treatment does not necessarily equate to ongoing transmission potential, as these parasites may have been rendered non-viable by the primaquine dose [40]. Also, the sizable anemia prevalence in our participants, 30% on enrolment, might have contributed to the delayed clearance observed. Additionally, the high initial parasite load in 56.1%, over half of our participants (geometric mean of 13,513 parasites/μL) could contribute to delayed clearance and increased risks of treatment failure.

The association between drug efficacy and parasite biomass is multifactorial and can be influenced by various factors such as hemoglobin level, host immune response, drug resistance, and individual variations. The higher parasite biomass during drug initiation can affect the rate of parasite clearance and increase risks of tolerance, which is an important measure of drug efficacy [41]. Although Chloroquine (CQ) was withdrawn as the first-line treatment for *P. falciparum* over two decades ago, its continued use for *P. vivax* might exerts indirect selection pressure on co-endemic *P. falciparum* populations. This sustained pressure may maintain the prevalence of CQ-resistance markers, complicating integrated malaria management strategies [42].

The diagnostic landscape in Western Ethiopia is further complicated by the emergence of *P. falciparum* histidine-rich protein 2 and 3 (*pfhp2*/3) gene deletions. A recent study in the nearby Assosa zone reported a high prevalence of these deletions, which can lead to false-negative RDT results [Alemayehu et al., 2021]. While our study utilized both microscopy and RDTs to ensure accurate enrollment, the reliance on RDTs alone in routine clinical settings in Bambasi could lead to significant under-diagnosis of falciparum malaria. This highlights the critical need for continued microscopy-based therapeutic efficacy surveillance to monitor both drug performance and diagnostic escape.

As one of the key elements of CQ resistance, Pfcrt-76T high prevalence has been observed in a previous study in Ethiopia, particularly in Adama, Metehara, and Olenchiti, where it was found in 95.7%, 92.5%, and 84.5% of isolates, respectively [42]. The Sudanese study that sequenced samples collected from 2016 to 2020 close to the Ethiopian border detected [43] *PfK13-*R622*I* mutation in 53.8% while in our study, the proportion was not assessed.

Strikingly, the efficacy was 93% in Sudan and 91.3% in Ethiopia, when the threshold for drug change is 90% or less as per WHO [19]. Moreover, the rapid increase in the proportion of detection of the *PfK13*-R662I in the region and broader is alarming. The Current study in five regions of Ethiopia reveals the proportion of R622I rose to 15.7% with additional previously validated by WHO as artemisinin resistance, particularly P675V in Sudanese refugee clinic in Gambella region of Ethiopia [44].

In our study, although the AL efficacy remains near the range of the WHO threshold, we suggest that the combination of a high proportion of MOI and the observed high prevalence of residual submicroscopic parasitemia after ACT treatment may contribute to the decline in efficacy.

As we found high MOI, which aligned with other single nucleotide point mutations, can lead to drug resistance and treatment failure, as different parasite genotypes may have varying susceptibility to antimalarial drugs [45]. Following this, the malaria elimination and control program may be hindered due to the high prevalence of MOI with high recombination rates, harboring multiple genetically distinct parasite clones in endemic settings leading to diverse parasite clone isolates, contributing to high production of gametocytes, rapid emergence, and distribution of drug resistant *P. falciparum* parasite strains that contribute lowering efficacy [46–48]. Notably, the PfK13-R622I mutation was initially identified in 2016 in northern Ethiopia, with 2.4% prevalence, which rose to 9.5% in 2018 [49], and a higher proportion (9.5%) was detected in the samples collected in 2018 from the same area [50] and rose 15.7% sample pooled from five regions of Ethiopia. A similar trend was observed in Eritrea, where the mutation increased from 8.6% in 2016 to 21.0% in 2019 [51]. This increasing trend in the proportion of *P. falciparum* strains harboring the PfK13-R622I mutation over a short period calls for an explicit understanding of the comparative advantage the mutation confers.

The combination of AL plus SLD PQs showed a better effect in reducing the gametocyte carriage and successful clearance of asexual parasites [39]. On the other hand, the duration of gametocyte clearance was long in our study; of the 10 participants with detectable gametocytes, 1 remained gametocytemic at day 3, and complete clearance was achieved by day 7. Given that the efficacy observed is alarmingly close to the WHO threshold of 90%, our study suggests that the combination of high MOI, high density of pretreatment parasite, and residual parasitemia may contribute to declining efficacy rates.

### Study limitations

Our study has three primary limitations. First, pharmacokinetic monitoring and in vitro susceptibility testing were not performed, which are critical for distinguishing between parasite resistance and sub-therapeutic drug exposure. Second, the absence of *Pfkelch13* and other molecular marker sequencing (such as *Pfmdr1* and *Pfcrt*) hinders the direct attribution of the observed 91.3% efficacy and the 16.9% Day 3 parasite positivity to specific genetic mutations. Consequently, we cannot confirm if the observed decline in artemether-lumefantrine effectiveness is driven by the emergence of artemisinin partial resistance or partner-drug tolerance in this setting. Finally, the study was not powered to evaluate gametocyte clearance, limiting conclusions about PQ’s transmission-blocking efficacy.

## Conclusions

The PPA CE-corrected efficacy of AL + PQ was 91.3% (Table 4). Although this remains just above the WHO’s 90% threshold for policy change, it indicates a potential decline in AL’s effectiveness in this high transmission setting. High MOI, pretreatment parasite density, and potential residual submicroscopic parasitemia may contribute to this reduced efficacy. Continuous monitoring using advanced molecular approaches, such as next-generation sequencing (NGS), is essential to detect resistance-associated mutations early and inform Ethiopia’s malaria treatment policies. Strengthened surveillance is critical to sustain malaria control progress in the study area and beyond.

## Supporting information

S1 Fig Trends of Confirmed Malaria Cases in Ethiopia (2015–2023). (DOCX)

S2 Fig Spatiotemporal trends in the prevalence of the PfK13-R662I mutation in the Horn of Africa (2016–2020). The bar chart illustrates the rapid regional emergence of the artemisinin partial resistance marker, *PfK13-R662I*, across Ethiopia, Eritrea, and Sudan. Prevalence data and denominators are derived from the following regional surveillance studies: Ethiopia 2016 (*n* = 410) [40]; Ethiopia 2018 (*n* = 350) [41]; Eritrea 2018 (*n* = 150) [42]; Eritrea 2019 (*n* = 200) [42]; and Sudan 2016–2020 (*n* = 120) [34]. The steady upward trend of this mutation in regions bordering the current study site provides a plausible biological context for the observed 91.3% therapeutic efficacy of artemether-lumefantrine in Western Ethiopia.
(DOCX)

S3 Fig Individual Asexual parasite clearance trajectories (Day 0–Day 3). The spaghetti plot illustrates the *log*10 -transformed asexual parasite density for each of the 123 participants (blue lines). The thick red line represents the mean density decline for the cohort. The vertical axis is presented on a logarithmic scale to highlight the magnitude of parasite reduction. The consistent downward trajectory across all participants, including those with high baseline densities (>100,000 parasites/μL), demonstrates the rapid parasitological knockdown achieved by the artemether-lumefantrine plus primaquine regimen.
(DOCX)

S4 Fig Trend Statement Checklist: A comprehensive checklist summarizing the key components in designing a non-randomized clinical trial, highlighting participant selection, intervention details, follow up, informed consent and participant safety, reporting result and documentation, outcome measures, along with a discussion on the implications for future research. (DOCX)

S1 File Study protocol: anti-malaria drug efficacy studies (TES). (DOC)
