# Supplementary figures and images for: Therapeutic efficacy of artemether-lumefantrine plus single low dose primaquine for the treatment of uncomplicated Plasmodium falciparum malaria in a high transmission setting, Western Ethiopia
Source: PLoS One. 2026 Jul 17;21(7):e0335833. doi: 10.1371/journal.pone.0335833 (PMC13379081; doi:10.1371/journal.pone.0335833)

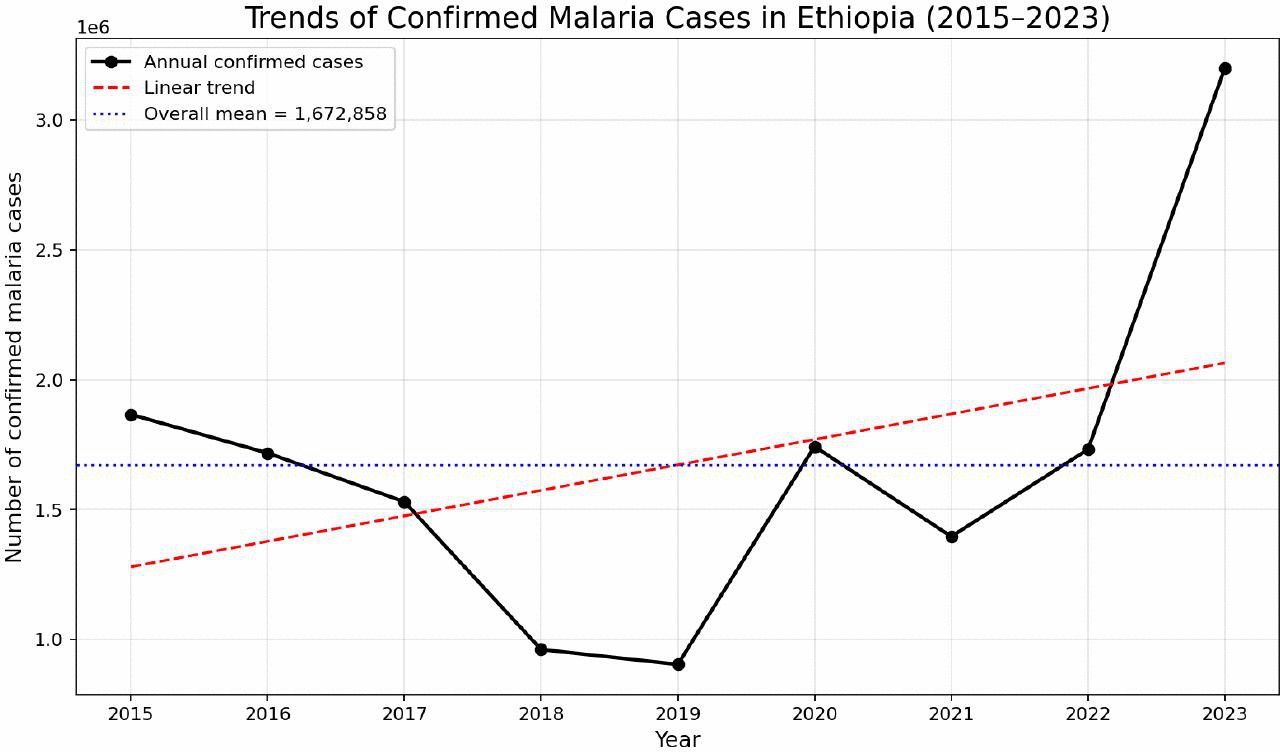


**S1 Fig 1. Trends of Confirmed Malaria Cases in Ethiopia (2015-2023).**

Supplement: S1 Fig — (DOCX) [file pone.0335833.s001.docx]

**
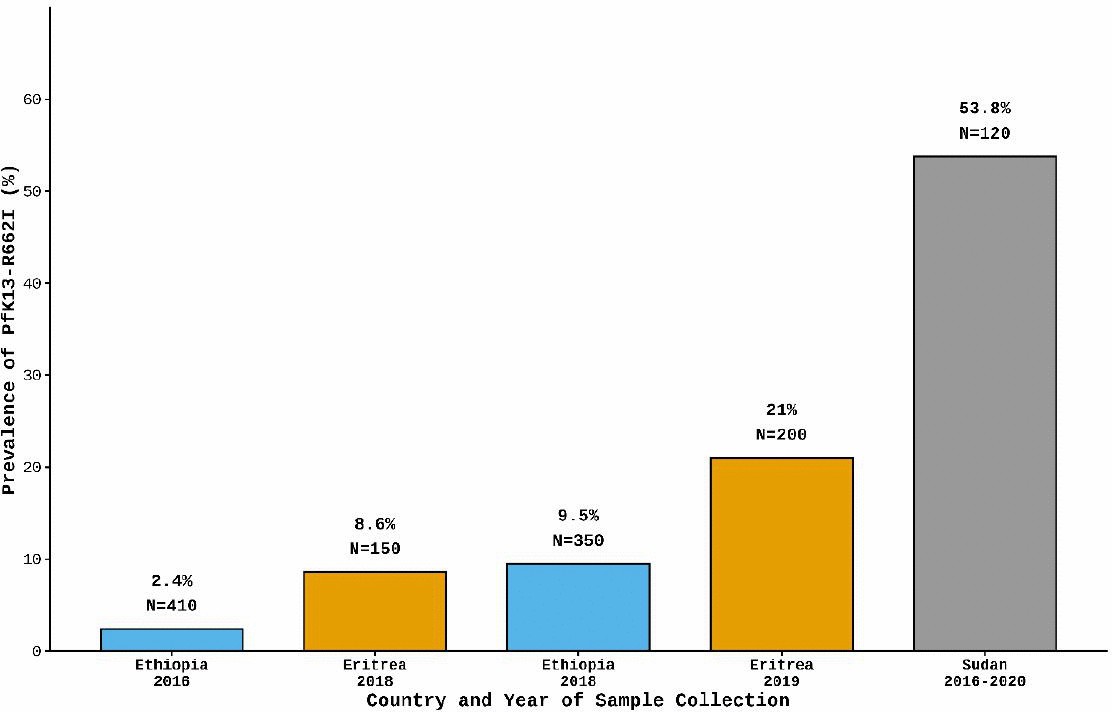
**

**S2 Fig 2. Spatiotemporal trends in the prevalence of the *PfK13-R662I* mutation in the Horn of Africa (2016–2020).**

Supplement: S2 Fig — The bar chart illustrates the rapid regional emergence of the artemisinin partial resistance marker, PfK13-R662I, across Ethiopia, Eritrea, and Sudan. Prevalence data and denominators are derived from the following regional surveillance studies: Ethiopia 2016 (n = 410) [40]; Ethiopia 2018 (n = 350) [41]; Eritrea 2018 (n = 150) [42]; Eritrea 2019 (n = 200) [42]; and Sudan 2016–2020 (n = 120) [34]. The steady upward trend of this mutation in regions bordering the current study site provides a plausible biological context for the observed 91.3% therapeutic efficacy of artemether-lumefantrine in Western Ethiopia. (DOCX) [file pone.0335833.s002.docx]
