# Supplementary material for: Therapeutic efficacy of artemether-lumefantrine plus single low dose primaquine for the treatment of uncomplicated Plasmodium falciparum malaria in a high transmission setting, Western Ethiopia
Source: PLoS One. 2026 Jul 17;21(7):e0335833. doi: 10.1371/journal.pone.0335833 (PMC13379081; doi:10.1371/journal.pone.0335833)
Supplement: S3 Fig — The spaghetti plot illustrates the log10 -transformed asexual parasite density for each of the 123 participants (blue lines). The thick red line represents the mean density decline for the cohort. The vertical axis is presented on a logarithmic scale to highlight the magnitude of parasite reduction. The consistent downward trajectory across all participants, including those with high baseline densities (>100,000 parasites/μL), demonstrates the rapid parasitological knockdown achieved by the artemether-lumefantrine plus primaquine regimen. (DOCX) [file pone.0335833.s003.docx]

**Supplementary 3 Figure 3 (S3 Fig.3)**

**
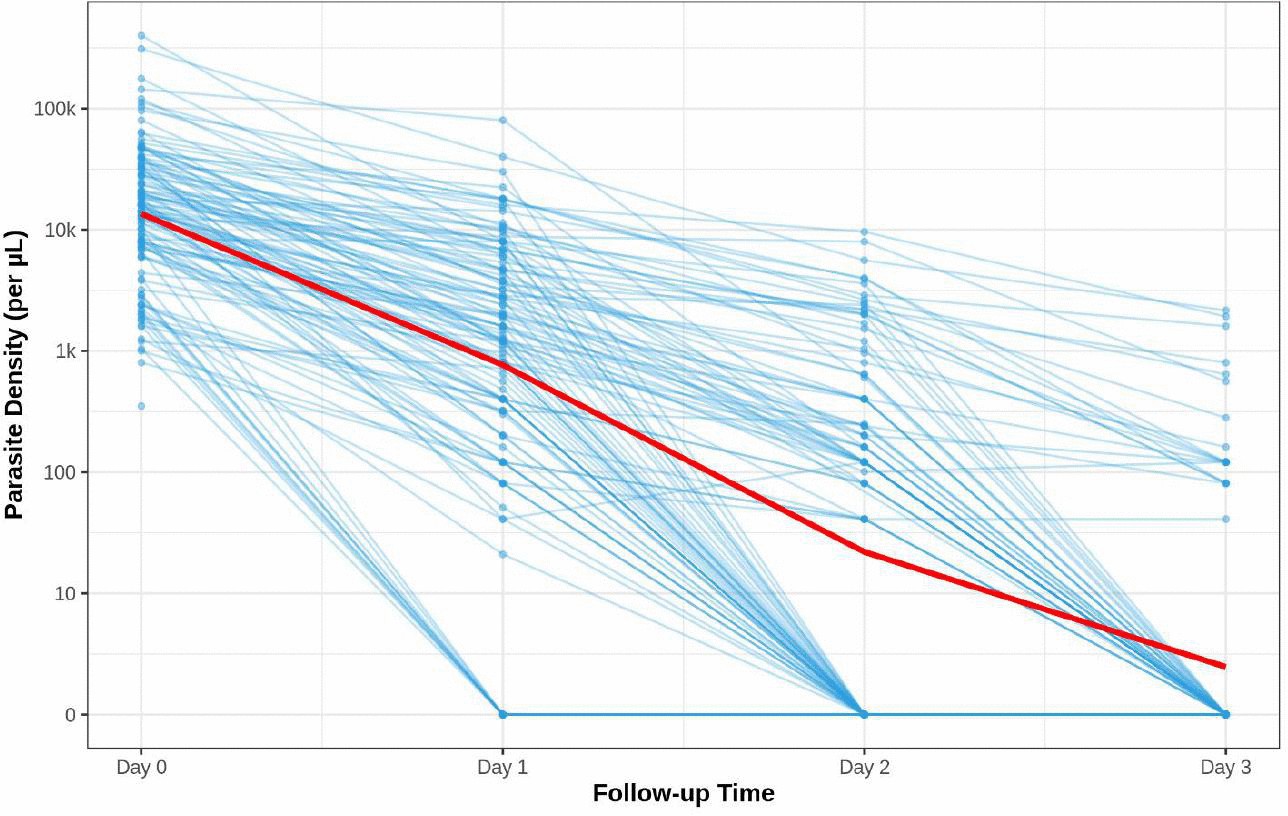
**

**S3 Fig. 3 Individual asexual parasite clearance trajectories (Day 0–Day 3).** The spaghetti plot illustrates the *log*10​-transformed asexual parasite density for each of the 123 participants (blue lines). The thick red line represents the mean density decline for the cohort. The vertical axis is presented on a logarithmic scale to highlight the magnitude of parasite reduction. The consistent downward trajectory across all participants, including those with high baseline densities (>100,000 parasites/μL), demonstrates the rapid parasitological knockdown achieved by the artemether-lumefantrine plus primaquine regimen.
