# Supplementary material for: Therapeutic efficacy of artemether-lumefantrine plus single low dose primaquine for the treatment of uncomplicated Plasmodium falciparum malaria in a high transmission setting, Western Ethiopia
Source: PLoS One. 2026 Jul 17;21(7):e0335833. doi: 10.1371/journal.pone.0335833 (PMC13379081; doi:10.1371/journal.pone.0335833)
Supplement: S1 File — (DOC) [file pone.0335833.s005.DOC]

**Ethiopia *in-vivo* efficacy study 2019: Therapeutic efficacy of Artemether-Lumefantrine plus Single Dose Primaquine for the treatment of uncomplicated *Plasmodium falciparum* and Chloroquine plus 14 days Primaquine for uncomplicated *Plasmodium vivax*.**

COORDINATED BY:

1. Armauer Hansen Research Institute, Addis Ababa
2. Federal Ministry of Health, Addis Ababa,
3. ICAP at Columbia University

# Table of Contents

[Table of Contents I](#__RefHeading___Toc16495082)

[SUMMARY III](#__RefHeading___Toc16495083)

[1. BACKGROUND 1](#__RefHeading___Toc16495084)

[2. STUDY DRUGS: 2](#__RefHeading___Toc16495085)

[2.1 Artemether-Lumefantrine (Coartem or AL) 2](#__RefHeading___Toc16495086)

[2.2 Chloroquine (CQ- as sulfate): 2](#__RefHeading___Toc16495087)

[2.3 Primaquine (PQ): 3](#__RefHeading___Toc16495088)

[*3* OBJECTIVES 3](#__RefHeading___Toc16495089)

[3.1 Specific Objectives 3](#__RefHeading___Toc16495090)

[3.2 Secondary Objectives 3](#__RefHeading___Toc16495091)

[4. METHODS 4](#__RefHeading___Toc16495092)

[4.1 Study Design 4](#__RefHeading___Toc16495093)

[4.2 Study Population 4](#__RefHeading___Toc16495094)

[4.3 Timing and duration of study 4](#__RefHeading___Toc16495095)

[4.3.1 Inclusion criteria 4](#__RefHeading___Toc16495096)

[4.3.2 Exclusion criteria 5](#__RefHeading___Toc16495097)

[4.4 Loss to follow-up 5](#__RefHeading___Toc16495098)

[4.5 Patient discontinuation or protocol violation 6](#__RefHeading___Toc16495099)

[5. TREATMENT 7](#__RefHeading___Toc16495100)

[5.1. Antimalarial Treatment 7](#__RefHeading___Toc16495101)

[5.2 Concomitant Treatment 7](#__RefHeading___Toc16495102)

[5.3 Rescue treatments for unexpected incidents 8](#__RefHeading___Toc16495103)

[5 EVALUATION CRITERIA 9](#__RefHeading___Toc16495104)

[6.1 Efficacy and safety evaluation 9](#__RefHeading___Toc16495105)

[*6.1.1* *Clinical efficacy endpoint* 9](#__RefHeading___Toc16495106)

[*6.1.3* *Safety end-points* 9](#__RefHeading___Toc16495107)

[6.2 Clinical Evaluation 9](#__RefHeading___Toc16495108)

[*6.3* *Laboratory Examination* 10](#__RefHeading___Toc16495109)

[7.1 Screening 12](#__RefHeading___Toc16495110)

[7.2 Enrollment and Follow-up 13](#__RefHeading___Toc16495111)

[9 Data Analysis Plan 15](#__RefHeading___Toc16495112)

[10 Minimum Sample Size 16](#__RefHeading___Toc16495113)

[12 Amendments to the Protocol 16](#__RefHeading___Toc16495114)

[13 IMPLEMENTATION OF THE PROJECT 17](#__RefHeading___Toc16495115)

[16. FACILITIES AVAILABLE FOR THE STUDY 22](#__RefHeading___Toc16495116)

[17. REFERENCES 23](#__RefHeading___Toc16495117)

[ANNEXES 4: Consent and Assent Forms 27](#__RefHeading___Toc16495118)

AHRI/ALERT Ethics Review Committee

Subject: ***Declaration sheet to guarantee the safety and proper care of study participants***

Title: *Ethiopia in-vivo efficacy study 2019: Therapeutic efficacy of AL -Single low Dose PQ for the treatment of uncomplicated falciparum malaria and CQ -14 days PQ for uncomplicated vivax malaria.*

**List of investigators, their affiliation and role:**

| Full Name | **Affiliation** | **Role** |  |
| --- | --- | --- | --- |
| Endalamaw Gadisa | AHRI | PI |  |
| Fitsum Girma | AHRI | Co-Investigator |  |
| Abebe Genetu | AHRI | Co-Investigator |  |
| Sinkinesh Behaksira | AHRI | Co-Investigator |  |
| Girma Shumie | AHRI | Co-PI |  |
| Jimma Dinsa | AHRI | Co-Investigator |  |

We hereby declare that, we will abide to the procedures approved by the ethics committee(s) to make sure that all safety procedures are adhered. We will make sure that the information sheet is communicated well in the language that participants understand. All personal data from the participants be kept confidential and be summarized anonymously.

On behave of the Investigators team

# SUMMARY

| **Title** | *Ethiopia in-vivo efficacy study 2019: Therapeutic efficacy of AL plus Single low Dose PQ for the treatment of uncomplicated falciparum malaria and CQ plus 14 days PQ for uncomplicated vivax malaria..* |
| --- | --- |
| **Study design** | Pprospective longitudinal surveillance |
| **Aims** | **Aim:** assess the therapeutic and transmission blocking efficacy of AL plus single dose PQ for uncomplicated Pf and CQ plus 14 days PQ for uncomplicated Pv infections  **Primary Aims:**   1. To measure the clinical and parasitological efficacy of AL + single dose PQ in patients above 6 months of age with uncomplicated Pf malaria. 2. To assess the transmission blocking effect of AL + single dose PQ as measure by rate of gametocyte clearance by RT-PCR 3. To measure the clinical and parasitological efficacy of CQ plus 14-days PQ in uncomplicated Pv malaria patients aged more than 6 months 4. To assess the transmission blocking effect of CQ + 14-days PQ as measure by rate of gametocyte clearance by RT-PCR 5. To differentiate recrudescence from new infection by molecular methods 6. To evaluate the incidence of adverse events |
|  | **Secondary Aims** |
|  | 1. To determine the polymorphism for known and/or novel molecular markers of resistance 2. To determine the blood concentration of CQ. 3. Determine parasite clearance rate, and fever clearance rate 4. To assess hematological response |
| **Study site** | Bambasi Health Centers |
| **Time frame** | The study will be conducted in main malaria transmission seasons between Sept. 2019 to Dec. 2019 |
| **Intervention** | AL plus single dose PQ (0.25mg/kg daily) for *Pf* cases and CQ plus 14 days of PQ (0.25mg/kg daily) for *Pv* cases |
| **Evaluation methods** | 1. Microscopic evaluation of thin and thick blood smears for plasmodium: adult and developmental stages and species 2. RT-nPCR techniques to assess for parasitemia, and gametocyte 3. nPRC-RFLP and/or sequencing to monitor known/novel drug resistance marker 4. Clinical and hematological methods for symptom and hemoglobin changes 5. HPLC-MS/MS  drug blood concentration |
| **Sample size** | 88 patients per treatment group (AL-PQ and CQ-PQ) for a total of 176 |
| **1^o^ outcome** | Therapeutic efficacy of AL-PQ for *Pf* and CQ-PQ for *PV* |
| **2^0^ outcomes** | 1. Parasite clearance rate between the treatment group as measured by microscopy adjusted by 18S based nPCR 2. Document on the transmission blocking potential of PQ bas measured by male gametocyte clearance rate 3. Document on genetic changes implicated in antimalarial drug resistance 4. Document on Adherence to drug regimen 5. Prevalence of serious adverse events (SAEs) related to treatment |
| **Funding** | FMOH through SDGs and GF |

1^o^ and 2^o^, means primary and secondary outcomes respectively;

# BACKGROUND

The World Health Organization (WHO) noted a slowdown of success in the fight against malaria during the last three years ^1^. The reported 219 million cases of malaria in 2017 exceeding the preceding years report by 2 million cases. During the same year, it claimed the life of 435 000 people, most of WHO African region accounted for 93% ^1^. In contrast, Ethiopia succeeded to further cut the malaria burden from the 2016 baseline by 240, 000 cases and is on track to meet the 2020 target. Moreover, Ethiopia is on a move to embark on phased approach to eliminate the disease by 2030 from the country ^2^. Malaria elimination is unlikely to be achieved by further up scaling of conventional control measures ^3^. Recent calls for malaria eradication have accepted that this problem will only be solved by combining and deploying different interventions that can act synergistically. One such class of interventions that is considered to be essential to the global eradication campaign includes therapeutics that prevents parasite transmission from the human host to the mosquito. These transmission-blocking therapies target the parasite at a critical population bottleneck when it is most vulnerable. Such treatment prevents the onward spread of the disease, thus limit the occurrence of new cases of infection ^4^.

One of the cornerstones of the current approach to malaria control is the provision of prompt, effective malaria treatment ^5^. Transmission reduction is now a key component of global efforts to control and eliminate malaria ^6^. The success of malaria elimination strategies will fundamentally rest on interrupting the cycle of parasite transmission between the Anopheles mosquito vector and humans ^7^. Disruption of transmission could be achieved by focusing on synchronized strategies targeting not only transmission from mosquito to human but also from human to mosquito, prompting renewed interest both in so-called “transmission-blocking” human malaria vaccines and the role of antimalarial drugs for preventing transmission to mosquitoes ^7; 8^. Therefore, transmission-reducing potential of combinations of ACTs and CQ with gametocytocidal PQ, has gained the attention of the Ethiopian national program ^2^.

As chemotherapy remains the mainstay, surveillance of therapeutic efficacy over time become an essential component of malaria control/elimination efforts. The endeavor ensures to deliver effective and safe treatment, and revise national drug policies when entailed. Hence, the analysis of drug response is a step towards understanding the profiles, treatment failures and adverse drug reactions ^9^. Furthermore, effectiveness of this combination is not yet well known. Therefore, new approaches that efficiently avert relapses and radical cure as well as gametocyte clearance and prevent recurrence need to be evaluated.

As the intention is also transmission blocking; efficacy trials involving radical cures should also consider gametocyte clearance tests in addition to the usual *in vivo* efficacy. The proportion of infections carrying gametocytes and gametocyte density are frequently reported along with estimates for asexual parasite density in population-based studies as a surrogate marker for human-to-mosquito transmission potential ^10^. However, microscopic evidence of gametocytaemia may not necessarily be as accurate an indicator of human-to-mosquito transmissibility as might be expected ^7; 11; 12^. Observations in endemic areas indicated that gametocytes often circulate at densities close to the threshold of microscopic detection ^13; 14^. Molecular techniques that detect particularly very low gametocyte densities that escape detection by light microscopy hence are necessary ^15; 16^.

Further exploration of antimalarial drug resistance genetic marker showed that in African endemic settings a decline in the prevalence of *Pfmdr1*-86Y and *Pfmdr1*-1246Y mutations during the last ten years. The phenomenon was shown to be associated with a reduced sensitivity to AL and increase in the sensitivity to Artesunate-Amodiaquine ^17^. Such observations thus have implications for planning strategies to cycle drugs or use multiple first-line therapies to maintain drug efficacy consolidating the need to involve molecular tools to detect drug resistance marker.

Ethiopia has been introduced, AL + Single dose PQ for the treatment of uncomplicated *P. falciparum and CQ+ 14d PQ for treatment of P.vivax malaria* since 2016.However, no study has been done, Therefore, the aim of this study was to assess treatment outcome of the recent WHO recommendation of adding a single PQ dose (0.25 mg/kg) to AL and CQ +14d PQ regimen for treatment of uncomplicated *P. falciparum and P.vivax malaria* in Ethiopia

# STUDY DRUGS:

The following are the known efficacy, adverse effects, and pharmacokinetics of treatment drugs that will be used in the study are *registered antimalarials approved and recommended first lines as per the national guideline.*

- 1. Artemether-Lumefantrine (Coartem or AL)***:*** *AL is currently the first-line anti-malarial recommended by Ethiopia FMOH for the treatment of uncomplicated falciparum malaria. Adverse events are generally mild, most commonly GI (vomiting and diarrhea) and hematologic (anemia and eosinophilia) ^18^. Its safety was demonstrated in studies from Ethiopia and Uganda ^19; 20^. Artemether has a short half-life of 0.86-5.16 hours, while lumefantrine has a long elimination half-life of 32.7-275 hours ^21^*.
  2. Chloroquine (CQ- as sulfate): *CQ is the recommended 1^st^ line treatment of vivax*

*malaria. The side effects include dizziness, skeletal muscle weakness, mild gastrointestinal disturbances (nausea, vomiting, abdominal discomfort, and diarrhea), and pruritus ^22^. CQ has a long elimination half-life of 108-291 hours (4-12 days)* ^21^.

- 1. Primaquine (PQ): *Primaquine is an 8-aminoquinoline that is highly active against the liver and sexual stages of malaria parasites. GI discomfort is dose-related. Importantly, PQ, when used for radical cure, can cause life-threatening hemolysis in patients who are G6PD deficient. PQ is recommended as single 0.25mg/kg PQ* *with AL in falciparum and 14-days of PQ (0.25mg/kg daily)) in case of Pv^2^. Elimination time for PQ is 3.5-8 hours.*

# OBJECTIVES

To assess the therapeutic efficacy of AL plus single dose PQ for uncomplicated Pf and CQ plus 14 days PQ for uncomplicated Pv infections based on parasitological, clinical, molecular and hematological parameters.

## 3.1 Specific Objectives

1. To measure the clinical and parasitological efficacy of AL + single dose PQ in patients aged more than 6 months with uncomplicated Pf malaria.
2. To measure the gametocyte clearance of AL + single dose PQ as measure by PCR in patients aged more than 6 months years with uncomplicated Pf malaria
3. To measure the clinical and parasitological efficacy of CQ plus 14-days PQ in uncomplicated Pv malaria patients aged more than 6 months
4. To measure the gametocyte clearance of CQ + 14 days PQ as measure by PCR in uncomplicated Pv malaria patients aged more than 6 months years,
5. To differentiate recrudescence from new infection by molecular methods
6. To evaluate the incidence of adverse events

## 3.2 Secondary Objectives

1. To determine the polymorphism for known molecular markers of resistance
2. To determine the blood concentration of CQ.
3. Determine parasite clearance rate, and fever clearance rate
4. To assess hematological response

# 4. METHODS

## 4.1 Study Design

A prospective longitudinal surveillance study design will be used. Self-presenting febrile (at time of presentation or in the past 48 hours) patients with uncomplicated *P. falciparum* or *P. vivax* mono-infections who meet the study inclusion criteria will be enrolled. Patients will be treated with as per national treatment guidelines of Ethiopia ^2^. Clinical and parasitological parameters will be monitored over a 42-day follow-up period. The follow-up will be on a fixed schedule for check-up visits and corresponding clinical and laboratory examinations. Upon recruitment confirmatory diagnosis will be done by microscopy or MultiSpp RDT. Vinous blood (3ml) in EDTA coated tubes (2ml) for molecular and hemoglobin (Haemocue, Sweden), and in RNA protect (1ml) for gametocyte detection and quantification will be collected on the follow up days (0, 2, 3, 7, 10, 14, 21, 28, 42).

Study Sites

Based on the available data of malaria cases, Bambasi.

## 4.2 Study Population

The population of interest consists of patients 6 months of age and above living within 20km radius of the health center and diagnosed with uncomplicated Pf or PV malaria. A written informed consent will be sought from participants 18 years and above. For minors, <18 years of age, parental/guardian written informed consents will be obtained. For children between the age of 11 and 17 years verbal assent will be taken in addition to their parent/guardians’ informed written consent.

## 4.3 Timing and duration of study

The study will be conducted during the major transmission season, September to December, 2019.

### 4.3.1 Inclusion criteria

1. Those living within the catchment of the health facility with traceable address
2. Volunteer to give their address and willing to not to travel for the duration of the study
3. Volunteer to comply with the study protocol; schedule visit or get tracked in case defaulted
4. Age ≥ 6 months
5. Slide-confirmed infection with Pf, with parasitemia of 500-100,000 asexual forms/μl or slide confirmed infection with *P. vivax* with > 250 asexual forms/μl
6. Axillary temperature ≥ 37.5º C or history of fever during the previous 24 or 48 hours for Pf and Pv infection, respectively
7. Ability to swallow oral medication

### 4.3.2 Exclusion criteria

1. General danger signs or symptoms of severe malaria (see Annex II)
2. Mixed plasmodium infection
3. Severe anemia, defined as hemoglobin (Hb) < 5 g/dl
4. Presence of febrile conditions caused by diseases other than malaria (e.g. measles, acute lower respiratory tract infection, severe diarrhea with dehydration)
5. Serious or chronic medical condition (e.g. cardiac, renal, hepatic diseases, sickle cell disease, HIV/AIDS)
6. Positive pregnancy test or breastfeeding
7. Refusal to take pregnancy test (women of child-bearing age = 12–49 years old or menstruating)
8. Unable or unwilling to take contraceptives for women of child-bearing age
9. History of hypersensitivity to test medication or used as rescue treatment in this study
10. Taking regular medication which may interfere with antimalarial pharmacokinetics or efficacy (see Annex IV)
11. Children weighing less than 5 kilograms
12. Severe malnutrition in child aged between 6-60 months as defined by presence of symmetrical oedema involving at least the feet or has a mid-upper arm circumference < 115 mm

## 4.4 Loss to follow-up

Loss to follow-up occurs when, despite all reasonable efforts, an enrolled patient does not attend the scheduled visits and cannot be found. No treat­ment outcome will be assigned to these patients. Every effort must be made to schedule a follow-up visit for patients who fail to return to the study site, especially after administration of the study drug. If these patients cannot be found, they will be classified as lost to follow-up and censored or excluded from the analysis. Patients who are lost to follow-up but who subsequently return to the study site before day 42 will not be turned away and will be encouraged to return for check-up visits. The principal investigator will decide whether the patient is to be definitely classified as lost to follow-up on the basis of his or her history or is to be maintained for the analysis as per the standard operating procedures.

## 4.5 Patient discontinuation or protocol violation

Patients meeting any of the following criteria will be withdrawn:

- Withdrawal of consent
- Failure to complete the treatment
  - Vomiting both initial and replacement doses at any single time in the treatment (i.e. if the patient vomits both attempts to administer the morning dose that would require withdrawal; however, vomiting the initial morning dose but not the morning replacement dose would not require withdrawal), persistent vomiting
  - Severe side-effects necessitating hospitalization
  - Progression to severe malaria
  - Failure to attend the scheduled visit during the first 3 days
- Enrolment violation
  - Severe malaria on day 0
  - Erroneous inclusion of a patient outside of the inclusion/exclusion criteria
- Voluntary protocol violation: Antimalarial (or antibiotics with antimalarial activity) treatment administered by a third party or self-medication with antimalarial (or antibiotics with antimalarial activity) assessed by asking participants or their caretaker at follow-up visits (Annex IV)
- Involuntary protocol violation
  - Occurrence during the follow-up of concomitant disease that would interfere with a clear classification of the treatment outcome
  - Detection of a mono-infection with another malaria species during follow-up
  - Misclassification of a patient due to a laboratory error (parasitemia) leading to the administration of the rescue treatment

# 5. TREATMENT

## Antimalarial Treatment

All study drugs will be obtained from WHO Global Malaria Program. Participants enrolled in this study will be treated as per the 4^th^ edition of the national malaria guideline ^23^. The correct drug doses will be as indicated in the aforementioned guideline ***page 104 to 107***, and summarized on the dosing chart of Annex V this protocol. Participants with confirmed Pf will be treated with AL plus single dose PQ; six-dose regimen of Coartem® given twice daily for three consecutive days and single low dose PQ (0.25 mg/Kg body weight). On days 0, 1 and 2, the first doses of the medication will be administered in the study health center under direct supervision by the study team (nurse), while the second dose of day 0 medication will be administered 8 hours later and, on day 1 and day 2, 12 hours later, at home by the patient or caregiver. The medications to be given at home will be prepared by the study nurse during the clinical visit with proper and clear verbal instruction on when and how to take the medication.

NB: *All doses of medicine will be administered under the supervision of a quali­fied and trained study nurse. The patients will be observed for 30 min after dosed for adverse reactions or vomiting. Those who vomit during this observa­tion period will be re-treated with the same dose of medicine and observed for an additional 30 min. If the patient vomits again, he or she will be with­drawn and offered rescue therapy; and will be referred to higher level of care for management with parenteral artesunate therapy and withdrawn from the study. This is standard practice for anti-malarial treatment.*

## 5.2 Concomitant Treatment

Using standard procedures, health professionals will administer supportive treatment to patients as might be necessary: participants with admission axillary temperature >38^0^C will receive a standard dose of 10 mg per kilogram paracetamol tablets/syrup every 6 hours as needed until the next visit. *All patients enrolled in the study will be given two additional doses of paracetamol for use at home.* Patients or parents/guardians will be instructed in the use and application of tepid sponging and fanning.

## Rescue treatments for unexpected incidents

AL, CQ and PQ are part of the national treatment guideline. The common adverse events (AEs) associated with each drugs are:

- AL: dizziness, headache, anorexia, nausea and abdominal pain, noting that these events may also be attributable to malaria.
- CQ: blurred vision, nausea, vomiting, abdominal or stomach cramps, headache, diarrhea, temporary hair loss, changes in hair color, or muscle weakness.
- PQ: nausea, stomach pain or upset, vomiting, loss of appetite, heartburn, abdominal cramps and hemolysis in G6PD deficient patients. All adverse events will be assessed and recorded by the health staff at each site participants or parents/guardians of minors will be properly oriented on the use of the urine chart and advised to stop taking PQ and immediately report to the health facility. If needed, participants will be referred to the nearest referral link for management.

Study staff will be at the clinic 24 hours a day, including holidays. In the event of any serious adverse event (SAE) (life threatening or requiring hospitalization like difficulty breathing, convulsions, and change in mental status), the patient will be referred to the referral hospital for further management. Transportation to the hospital will be provided by the study. In addition, SAEs will be reported to responsible authorities, respective IRB’s and Data Safety Monitoring Board according to policies on incident reporting.

Participants or parents/guardians of participants will be counseled to immediately seek clinical care if they or their child develops a raised rash (urticaria) or difficulty breathing suggestive of a severe allergic reaction. These patients will be treated with diphenhydramine or chlorpheniramine and provided with oral quinine for seven days which is the 2^nd^ line malaria treatment as per national guidelines. Patients with signs of severe malaria (altered mental status, coma, convulsions, hemoglobin <5g/dL, respiratory distress, circulatory collapse, or abnormal bleeding), or persistent vomiting will be linked for hospitalization and treatment with artesunate and relevant supportive treatments according to national guidelines^23^.

# EVALUATION CRITERIA

## Efficacy and safety evaluation

The aim of PQ radical cure is to shorten the duration of gametocyte circulation and hence the probability of onward transmission of malaria therefore facilitating control and elimination efforts. The primary outcome of this study will be to monitor the efficacy of AL plus single dose PQ for Pf and CQ plus 14 days PQ for Pv as a routine follow up study in patients aged greater than 6 months and produce baseline data for the Ethiopian program on rate of gametocyte clearance using PQ radical cure

### *Clinical efficacy endpoint*

Clinical efficacy will be based on an assessment of the parasitological and clinical outcomes of antimalarial treatment according to the latest guidelines from WHO (3). Accordingly, all Pf and Pv patients will be classified as having an early treatment failure (ETF), late clinical failure (LCF), late parasitological failure (LPF), or adequate clinical and parasitological response (ACPR) (Annex I).

- - 1. ***Gametocyte clearance rate***

Different stages of the parasites including trophozoites, female and male gametocytes will be detected and quantified will be measured using microscopy and quantitative real time reverse transcriptase polymerase chain reaction (qRT-PCR) on samples collected on days 0, 2, 3, 7, 10, 14, 21, 28, and 42.

### *Safety end-points*

The incidence of any adverse event that entails the discontinuation of the medication: see section 5.2.

## Clinical Evaluation

Clinical assessment will be done on enrolment and the scheduled visit dates: Days 0, 1, 2, 3, 7, 14, 21, 28, 35, and 42. A standard physical examination with complete medical history, body temperature, demographic information, and contact details will be recorded at baseline. Body weight and body temperature will be taken at enrolment and subsequent visits. Appropriately calibrated weight scales and thermometer verified before the study begins and checked at regular intervals will be used to measure. Digital thermometer with a precision of 0.1 °C will be used to measure temperature. The same route of measurement will be used throughout the study to take body temperature. In case the read is less than 36 degree it will be repeated. Participants should remove excessive clothing and young children should only wear undergarments while being weighed. Weight will be recorded to the nearest kilogram. For under five children the left mid-arm circumference will be measure to the nearest 0.2 cM. And For indication of severe malnutrition, pitting oedema on the dorsal surface of both feet will be assessed by thumb pressure for 3 seconds.

### *Laboratory Examination*

- - 1. **Smear microscopy:** thick and thin blood smears will be taken from all participants and prepared on the same slide for detection of parasites at all time-points during the 42-day follow-up period. Two ul of blood and 6 ul of blood will be collected to prepare thin and thick blood film respectively. Two smears will be prepared; the first will be stained rapidly with 10% Giemsa for 10–15 min for initial screening and examined by light microscopy immediately. On day 0, in order to rapidly confirm adherence to the lowest parasite density considered for enrollment, initial screening of patients will be made in 10% Giemsa-stained thick film after counting at least 1 parasite for every 6-8 WBCs, which corresponds to approximately 1000 parasites/μl.

The second slide will be stained slowly with 3% Giemsa 45–60 min to be examined later by an experienced technician to provide definitive parasite count and speciation. The below formula will be used to determine parasite density.

| Parasite density (per µl) = number of parasites counted × (6000–8000) |
| --- |
| Number of leukocytes counted (approximately 200) |

Two experienced laboratory technicians will examine slides independently. Parasite densities will be calculated from thick blood smears by counting the number of asexual parasites against 200 WBC. If the count is <10 parasites/200 WBC, at least 500 WBC will be counted. A thick blood smear will be declared negative if no parasites are seen in 1000 WBCs and mixed infection has been excluded in 100 fields on thick film. Gametocytes will be detected and counted in thick film. Thin blood smears will be examined to determine parasite species. Gametocytes will be counted in thick blood films against 1000 WBCs.

Two qualified microscopists will read all the slides independently, and parasite densities will be calculated by averaging the two counts. Blood smears with discordant results and ≥400 parasites/µL (differences between the two microscopists in species diagnosis, in parasite density of > 50% or in the presence of parasites) will be re-examined by a third, independent microscopist, and parasite density will be calculated by averaging the two closest counts. Blood smears with discordant results and <400 parasites/µL (differences between the two microscopists in species diagnosis, in parasite density differences ≥10, or in the presence of parasites) will be re-examined by a third, independent microscopist, and parasite density will be calculated by averaging the two closest counts.

- - 1. **Hemoglobin:** Hemoglobin concentrations will be measured using a portable spectrophotometer (HemoCue®, Angelholm, Sweden), on days 0, 14, 28, and 42.

- - 1. **Pregnancy Test:**

Urine pregnancy test will be done for menstruate or those within 12-49 years of age before enrolment and day 42 or earlier on withdrawal from the study. Female participants of child-bearing age will be counseled and given barrier contraceptive based on her type of choice for the duration of the study. They will also be followed to determine the occurrence and outcome of the pregnancy. Generally, follow-up will be no longer than 6–8 weeks after the estimated delivery date. Any premature termination of preg­nancy will be reported. While pregnancy itself is not considered an adverse event or a serious adverse event, any complication of pregnancy or elective termination for medical reasons will be recorded as an adverse event or a serious adverse event. A spontaneous abortion is always considered a serious adverse event and will be reported as such.

- - 1. **Nucleic acid extraction and quantitative PCR:**

DNA will be extracted from 6mm diameter punches that will be treated with 20µl Proteinase K (QIAGEN) in a total volume of 200µl containing Tissue Lysis Buffer (QIAGEN) at 56^o^C overnight in a water-bath, followed by extraction using commercial DNA extraction kit (QIAGEN). Real-time quantitative (qPCR) for parasite detection will be performed by targeting the 18S small rRNA gene for *Pf* and *Pv* using primer and probe sequences described in ^24; 25^. *Pf* parasites will be quantified using standard curves generated from a serial dilution of NF54 ring stage parasites ^24^. *Pv* parasite quantification will be done using plasmid constructs to infer copy numbers as described before ^25^. Blood samples in RNA protect buffers will be used for extraction of RNA using the RNeasy Mini Kit (QIAGEN) for gametocyte quantification ^25-27^, gametocyte commitment and maturation assays, sex ratio estimation ^28^, asexual stage parasites detection ^29^, and expression level of regulators of the balance between reproduction and replication ^30-32^.

- - 1. **Drug levels:**

Blood sample spot collected on filter paper will be used to determine the blood concentration of chloroquine at Day 7 and at time of treatment failure. Specimens will be coded (participant identification number, day of follow-up, date). Standard methods will be used ^33-35^ and the tests will be done both at CDC/AHRI as might be required.

1. **Screening, enrollment and follow up**

Screening, enrolment and follow up for eligible patients at the study sites will be done by trained (GCP and protocol specific) health professionals:

## Screening

1. Febrile (body temperature ≥37.5^o^C) or a history of fever in the last 48 hours patients; older than 6 months and living within 20 km of the study health facility are eligible. Those volunteering will further be screened as per the national malaria guideline microscopy confirmed P. falciparum or P. vivax will be enrolled
2. Enrollment will be done by a staff member not involved in the clinical assessment of the participant to ensure that consent is freely given. Patients who meet the screening criteria and the patient or the parent or guardian signed the informed consent form will be assigned with a unique study number. This number will become the patient's identifier in all forms and blood samples from that patient for appropriate tracking every event/record for the case.
3. After obtaining informed consent; each participant will be weighed. For children less than 5 years, presence of pitting oedema will be assessed and the left mid-upper arm circumference will be measured.

## Enrollment and Follow-up

Those volunteers who fulfill the initial screening criteria will go through the enrolling process:

1. The health professionals at site of enrolment will check the participant for signs/symptoms of:
   1. Clinical malaria and make a microscopic/MSPP RDT confirmation from figure prick
   2. Febrile illness other than malaria; including but not limited to pneumonia, otitis media, tonsillitis, measles, chicken pox, abscesses and
   3. Severe disease/ presence any danger signs, as indicated in the exclusion criteria. Volunteers found to have any of these conditions will be treated as per the appropriate national guidelines, and if deemed necessary will be sent to the next referral the referral link in the health system.
   4. If the volunteer fulfills the inclusion criteria
      1. The approved CRFs will be completed for each.
      2. Their appointment schedule will be clearly explained, and a follow-up card with a personal identification number will be provided (see the study schedule (Annex VI) and

2. For every volunteering women of child bearing (12-49 years) or who are menstruating; a Urine Pregnancy Test will be conducted. And if tested positive will be excluded.

3. Those enrolled, confirmed *Pf or Pv cases*, will have a venous blood collected to assess for the following:

- - - - Thin smear will be used to verify parasite species and to conduct a formal parasite count. Two microscopists will independently read each smear; if the 2 readings are >50% discordant, a third expert microscopist will examine the slide and make the final decision.
- Hemoglobin measurement (Hemocue™ Hb201/301+; HemoCue, Angelholm, Sweden) will be carried out and those with hemoglobin <5g/dL will be excluded.
- Filter paper samples and RNA protect samples will be prepared for testing
  - drug levels.
    - - - nPCR based assay will be done for species confirmation, and gametocyte and parasite load determination.

4. All enrolled participants will be treated as per the national Malaria Guideline.

5. Clinical reassessments will be made on days 1, 2, 3, 7, 14, 21, 28, 35, and 42 (see Annex VI for a table of scheduled procedures). Due orientation will be given for those put on 14 days PQ on urine color chart monitoring: Participants will be advised to return immediately on *any* day during the follow-up period if symptoms return and/or if they notice darkening of their urine stop taking PQ.

6. If participants do not appear for scheduled follow-up, the study team will attempt to reach them at home. For follow-up visits on day 1 and 2 while the study drugs are being administered, the study staff will attempt to reach the patient by phone in addition to the home visit on that day. After day 2, those who fail to return on their scheduled day but return one day early or one day late may still be included in the analysis. After day 2, an attempt will be made to reach them on the day of their missed visit and again the next day. After day 3, if failed to return on their scheduled day but return one day early or one day late may still be included in the analysis.

1. **Data Management**

All data, socio-demographic, clinical and laboratory will be recorded as generated on CRFs designated for the specific purpose. Data will be collected and recorded at the health facility by trained study staff members. Laboratorians may complete brief reports on laboratory findings to share with clinicians who will transfer these results to the case report form of the appropriate patient. Site supervisors will check CRF data entry at the end of follow-up for completeness and accuracy of recording. Data will be verified for consistency with the source documents, or ascribe source of error if any and completeness by the data manager at AHRI. Any change or correction to a CRF should be dated and explained and should not obscure the original entry. All data management and double entry using the REDCap will be done at AHRI data management center.

Confidentiality of data will be maintained; the paper forms will be kept in a locked file cabinet and the databases have standard password system for all onsite and offsite backups. The all partners stated in this protocol will have access to curetted and decoded data, no report or data shared will have personal identifiers/clues that in any way link to a study participant.

## Data Analysis Plan

Cleaned and validated data will be analyzed using STATA and WHO drug efficacy tools.

1. The **per protocol analysis** will consider patients who were withdrawn from the study or who were lost to follow-up are excluded, and **survival analysis**, where all enrolled patients are included in the analysis until the last day before drop-out. In addition to voluntary withdrawals or treatment non-compliance, patients will be considered for the analysis as withdrawn if the PCR results are unclassifiable.
2. The final analysis will include:

- A description of the patients screened, and the distribution of the reasons for exclusion.
- A description of the patients included in the study.
- The proportion of patients lost to follow-up or withdrawn, with 95% confidence intervals and a list of reasons for withdrawal.
- The proportion of adverse events and serious adverse events in all the patients included in the study
- The PCR-adjusted and unadjusted proportion of ETF, LCF, LPF, and ACPR in the Pf arm at day 28 and day 42 with 95% confidence intervals using both methods of analysis.
- Both drug-level and PCR-adjusted and unadjusted proportions of treatment failures in each Pv arm at day 28 and day 42 with 95% confidence intervals using both methods of analysis
- Frequency of severe malaria, anemia, and hospitalizations by treatment arm
- Parasite and gametocyte clearance rate: proportion of patients with negative thick blood smears on days 1, 2, 3.
- Fever clearance rate: proportion of patients without objective fever (axillary temperature < 37.5°C) on days 1,2,3
- Gametocyte carriage rates: proportion of patients with gametocytes on days 0, 7, 14, 21, 28, 35, 42.
- Hematological recovery: Change in mean hemoglobin concentration from day 0 to 14, 28, 42.

## Minimum Sample Size

Sample size was calculated to yield point estimates of efficacy for each drug-species group. To generate point estimates of drug efficacy, measured by ACPR, we assumed an ACPR of 95% and a confidence interval of 10% so that the lower bound would still meet the WHO threshold of 90% ACPR. A total of 73 patients per arm (drug-species group) would be required; factoring in 20% loss to follow-up and protocol violation, 88 patients per treatment group will be 176 per site.

1. **Ethical Considerations**

Before the commencement of the study, letter of support will be obtained Federal Ministry of Health, Disease prevention and control directorate, National Malaria control team. Approval will be sought from institutional ethics review committees; AHRI/ALERT.

## Amendments to the Protocol

After the protocol has been ethically approved, no change will be made without the agreement of the principal investigator, the sponsor(s), and the concerned ethics review committees which reviewed the protocol.

# IMPLEMENTATION OF THE PROJECT

Schedule for the implementation of the study

| Activities | | **May,2019** | | | **June, 2019** | | | | **July, 2019** | | | | **August, 2019** | | | | **September, 2019** | | | | **October, 2019** | | | | **Nov, 2019** | |
| --- | --- | --- | --- | --- | --- | --- | --- | --- | --- | --- | --- | --- | --- | --- | --- | --- | --- | --- | --- | --- | --- | --- | --- | --- | --- | --- |
|  |  | Wk2 | Wk3 | Wk4 | Wk1 | Wk2 | Wk3 | Wk4 | Wk1 | Wk2 | Wk3 | Wk4 | Wk1 | Wk2 | Wk3 | Wk4 | Wk1 | Wk2 | Wk3 | Wk4 | Wk1 | Wk2 | Wk3 | Wk4 | W1/2 | W3/4 |
| PREPARATORY PHASE (INCLUDING PROTOCOL REVIEW) | Preparation of protocol and ethical review |  |  |  |  |  |  |  |  |  |  |  |  |  |  |  |  |  |  |  |  |  |  |  |  |  |
|  | Purchase of supplies |  |  |  |  |  |  |  |  |  |  |  |  |  |  |  |  |  |  |  |  |  |  |  |  |  |
|  | Document development |  |  |  |  |  |  |  |  |  |  |  |  |  |  |  |  |  |  |  |  |  |  |  |  |  |
|  | Study team preparation |  |  |  |  |  |  |  |  |  |  |  |  |  |  |  |  |  |  |  |  |  |  |  |  |  |
|  | Study site preparation |  |  |  |  |  |  |  |  |  |  |  |  |  |  |  |  |  |  |  |  |  |  |  |  |  |
|  | Logistics arrangement for the study |  |  |  |  |  |  |  |  |  |  |  |  |  |  |  |  |  |  |  |  |  |  |  |  |  |
| FIELD WORK | Training/orienting study team members |  |  |  |  |  |  |  |  |  |  |  |  |  |  |  |  |  |  |  |  |  |  |  |  |  |
|  | Data collection |  |  |  |  |  |  |  |  |  |  |  |  |  |  |  |  |  |  |  |  |  |  |  |  |  |
|  | Monitoring of study |  |  |  |  |  |  |  |  |  |  |  |  |  |  |  |  |  |  |  |  |  |  |  |  |  |
| ANALYSIS AND WRITE UP | Data entry and analysis |  |  |  |  |  |  |  |  |  |  |  |  |  |  |  |  |  |  |  |  |  |  |  |  |  |
|  | reporting |  |  |  |  |  |  |  |  |  |  |  |  |  |  |  |  |  |  |  |  |  |  |  |  |  |

1. **Financial breakdown**

| **Ethiopia in-vivo efficacy study 2019: Therapeutic efficacy of AL plus Single Dose PQ for the treatment of uncomplicated falciparum malaria and CQ plus 14 days PQ for uncomplicated vivax malaria.** | | | | | | |
| --- | --- | --- | --- | --- | --- | --- |
|
|
|  |  |  |  |  |  |  |
| S. No. | Items | Per site | unit price /site | total price/site | Number of sites | Total price /4 sites |
|  | A.Consumables |  |  |  |  |  |
| 1 | Microscope slides, frosted edge, (pack of 100) | 70 | 360 | 25200 | 4 | 100800 |
| 2 | Lens tissue (pack of 100) | 10 | 900 | 9000 | 4 | 36000 |
| 3 | Hemolancets (pack of 200) | 15 | 130 | 1950 | 4 | 7800 |
| 4 | Torniquetof 100 | 2 | 2500 | 5000 | 4 | 20000 |
| 5 | Swabs, alcohol (70%), bottle of 1 lit | 12 | 70 | 840 | 4 | 3360 |
| 6 | Gloves, disposable medium size box of 100 pairs | 70 | 180 | 12600 | 4 | 50400 |
| 7 | Gloves, disposable, large size, box of 100 pairs | 70 | 150 | 10500 | 4 | 42000 |
| 8 | Immersion oil, bottle of 50ml | 2 | 290 | 580 | 4 | 2320 |
| 9 | Giemsa stain stock solution, bottle of 500ml | 2 | 950 | 1900 | 4 | 7600 |
| 10 | Absolute methanol of 1 liter | 3 | 490 | 1470 | 4 | 5880 |
| 11 | Injection syringes with needle, 5ml, of 100 | 30 | 250 | 7500 | 4 | 30000 |
| 12 | Injection syringes with needle, 10 ml of 100 | 40 | 300 | 12000 | 4 | 48000 |
| 13 | EDTA test tube rack of 100 | 50 | 220 | 11000 | 4 | 44000 |
| 14 | Vacutainer needle of 50 | 100 | 150 | 15000 | 4 | 60000 |
| 15 | Vacutainer needle holder of 1000 | 2 | 150 | 300 | 4 | 1200 |
| 17 | Cotton roll pack of 100g | 10 | 20 | 200 | 4 | 800 |
| 18 | HCG Test Kit (50/pack) | 10 | 225 | 2250 | 4 | 9000 |
| 19 | Sealable plastic Bag, pack of 50 | 10 | 290 | 2900 | 4 | 11600 |
| 20 | Safety Box of 3 piece plastick container | 10 | 111 | 1110 | 4 | 4440 |
| 21 | Whatman 903 Card for DBS collection of 200 | 20 | 3640 | 72800 | 4 | 291200 |
| 22 | Slide box of 100 | 30 | 300 | 9000 | 4 | 36000 |
| 23 | 1.5ml Eppendorf tube (100/pack) | 30 | 275 | 8250 | 4 | 33000 |
| 24 | Slide tray | 10 | 180 | 1800 | 4 | 7200 |
| 25 | Bio-hazard box sharp container of 50 | 1 | 300 | 300 | 4 | 1200 |
| 26 | Claser for DBS of 200 | 2 | 845 | 1690 | 4 | 6760 |
| 27 | Lens tissue, pack of 100 | 5 | 300 | 1500 | 4 | 6000 |
| 28 | Bio-hazard bag 24", pack of 50 | 5 | 285 | 1425 | 4 | 5700 |
| 29 | Tea-spoons (plastic disposable) | 300 | 8 | 2400 | 4 | 9600 |
| 30 | Spatula, pk of 100 | 3 | 200 | 600 | 4 | 2400 |
| 31 | Plastic cups (disposable) | 300 | 15 | 4500 | 4 | 18000 |
| 32 | Tissue paper (roll) | 50 | 14.5 | 725 | 4 | 2900 |
| 33 | Sugar in kg | 4 | 30 | 120 | 4 | 480 |
| 34 | Distilled water in 1L | 20 | 30 | 600 | 4 | 2400 |
| 35 | Battery cell AA 1.5V for Hemocue 301 | 8 | 50 | 400 | 4 | 1600 |
| 36 | Racked tips, 20 μL/ 960 tips/10 racks of 96 | 10 | 2500 | 25000 | 4 | 100000 |
| 37 | Racked tips, 10-100 μL max 960tips in 10 racks of 96 | 3 | 4500 | 13500 | 4 | 54000 |
| 38 | Racked tips, 1000 μL/ 960tips /10 racks of 96 | 5 | 4350 | 21750 | 4 | 87000 |
| 39 | HB 301 Microcuvettes(200/pack) | 6 | 6500 | 39000 | 4 | 156000 |
| 40 | Bottled water(1/2 liter *172 participants*9 visits) | 1548 | 7 | 10836 | 4 | 43344 |
| 41 | Biscuits (1 pkt*172 participants*9 visits) | 1548 | 3.5 | 5418 | 4 | 21672 |
|  | Sub total |  |  | 407914 |  | **1631656** |
|  | B.PCR coponents for qpcr and npcr |  |  |  |  |  |
| 42 | Agarose gel 500 gm | 1 | 8000 | 8000 | 4 | 32000 |
| 43 | v-shaped pcr plate of 20 (10pkt/20Pc) | 10 | 2600 | 26000 | 4 | 104000 |
| 44 | pcr plate sealer of 100 (5 pkt/100Pc) | 5 | 4500 | 22500 | 4 | 90000 |
| 45 | RNA protect kitof 250ml | 6 | 8200 | 49200 | 4 | 196800 |
| 46 | TBE buffer 20X of 500ml | 3 | 1300 | 3900 | 4 | 15600 |
| 47 | DNA extraction kit for pcr | 2 | 8700 | 17400 | 4 | 69600 |
| 48 | 5X PCR Buffer of 1.5 ml | 30 | 1300 | 39000 | 4 | 156000 |
| 49 | 25 mM MgCl2 of 1.5 ml | 30 | 1200 | 36000 | 4 | 144000 |
| 50 | 25 mMdNTPs of 1ml each | 20 | 2813 | 56260 | 4 | 225040 |
| 51 | Forward primer rplu5 | 8 | 3000 | 24000 | 4 | 96000 |
| 52 | Reverse primer rplu6 | 8 | 3000 | 24000 | 4 | 96000 |
| 53 | Forward primer FAL1 | 6 | 2900 | 17400 | 4 | 69600 |
| 54 | Reverse primer FAL2 | 6 | 2900 | 17400 | 4 | 69600 |
| 55 | Forward primer rviv1 | 6 | 2900 | 17400 | 4 | 69600 |
| 56 | Reverse primer rviv2 | 6 | 2900 | 17400 | 4 | 69600 |
| 57 | Taq DNA Polymerase, 5 U/μl of 50un | 6 | 2940 | 17640 | 4 | 70560 |
| 58 | Water RNase/DNAse 1 liter | 5 | 3125 | 15625 | 4 | 62500 |
| 59 | TaqMan MM 2x | 4 | 12064 | 48256 | 4 | 193024 |
| 60 | Primer Forward | 6 | 2790 | 16740 | 4 | 66960 |
| 61 | Primer Reverse | 6 | 2790 | 16740 | 4 | 66960 |
| 62 | Probe | 5 | 4300 | 21500 | 4 | 86000 |
| 63 | Gel loading dye 6X | 10 | 1300 | 13000 | 4 | 52000 |
| 64 | Genotyping | 25 | 1200 | 30000 | 4 | 120000 |
|  | Sub total |  |  | 555361 |  | **2221444** |
|  | C.Stationeries |  |  |  |  |  |
| 65 | Permanent marker, fine tips | 4 | 20 | 80 | 4 | 320 |
| 66 | Pen of 12 | 10 | 60 | 600 | 4 | 2400 |
| 67 | Pencil of 12 | 10 | 12 | 120 | 4 | 480 |
| 68 | Stapler medium size | 2 | 150 | 300 | 4 | 1200 |
| 69 | Paper | 2 | 500 | 1000 | 4 | 4000 |
| 70 | Staplerwire of 100/pack | 3 | 300 | 900 | 4 | 3600 |
|  | Sub total |  |  | 3000 |  | **12000** |
|  | D.Human Power |  |  |  |  |  |
| 71 | General practionair | 2 | 5000 | 10000 | 4 | 40000 |
| 72 | Pharmacist | 2 | 3500 | 7000 | 4 | 28000 |
| 73 | Nurse/HO | 2 | 3500 | 7000 | 4 | 28000 |
| 74 | Lab technologist | 2 | 3500 | 7000 | 4 | 28000 |
| 75 | Slide reader | 2 | 3500 | 7000 | 4 | 28000 |
| 76 | Confirmation/Ckecker 20 birr/slide | 1 | 42240 | 42240 | 4 | 168960 |
| 77 | Supervisor | 1 | 15000 | 15000 | 4 | 60000 |
| 78 | Local facillitator | 1 | 2500 | 2500 | 4 | 10000 |
| 79 | Patient compensation | 176 | 100 | 17600 | 4 | 70400 |
|  | Sub Total |  |  | 115340 |  | **461360** |
|  | E.Transportation |  |  |  |  |  |
| 80 | Fuel (600Km/Month*3M*4Wks*7Km/L*20 birr/L) | 1 | 20571.42857 | 20571.42857 | 4 | 82285.71429 |
| 81 | Driver 450/day*24 days | 1 | 10800 | 10800 | 4 | 43200 |
| 82 | Perdiem superviser (450/day*24day | 1 | 10800 | 10800 | 4 | 43200 |
|  | Sub Total |  |  | 42171.42857 |  | **168685.7143** |
|  | F.communation |  |  |  |  |  |
| 83 | Mobile card for coordinator 100birr/month*4 sites*3Months | 1 | 300 | 300 | 4 | 1200 |
| 84 | E-video for data transfer 750birr/month*3 months | 1 | 2250 | 2250 | 4 | 9000 |
|  | **Sub Total** |  |  | 2550 |  | **10200** |
|  | G Training |  |  |  |  |  |
| 85 | Refresher 12 trainees & 4 trainers/site* 150 birr/day * 3 days | 1 | 7200 | 7200 | 4 | 28800 |
| 86 | Perdiem [12 trainees & 4 trainers/site* 450 birr/day * 3 days] | 1 | 21600 | 21600 | 4 | 86400 |
|  | Sub total |  |  | 28800 |  | **115200** |
|  | over all total |  |  | 747222.4286 |  | 4620545.714 |
|  | 5% contingency |  |  |  |  | 231027.2857 |
|  | **Estimated budget in Ethiopian Birr for four sites** |  |  |  |  | **4591573** |
|  | **Estimated budget in USD for four sites** |  |  |  |  | **160040.8853** |

**NB:**

- *The budget assumes drugs will be made available by the program. Except for the supervisor and tracers all will be employees of the health system with top up for extra efforts.*

# FACILITIES AVAILABLE FOR THE STUDY

- Health facilities at the respective study sites within 24 hours access to the main study center: Bambasi.

# REFERENCES

1. WHO. (2018). World Malaria Report 2018. . In. (World Health Organization.

2. FMoH. (2018). National Malaria Guidelines. In, Disease, Prevention, andControl, eds. (Addis Ababa, Ethiopian Federal Ministry of Health

3. Griffin, J.T., Hollingsworth, T.D., Okell, L.C., Churcher, T.S., White, M., Hinsley, W., Bousema, T., Drakeley, C.J., Ferguson, N.M., and Basáñez, M.-G. (2010). Reducing Plasmodium falciparum malaria transmission in Africa: a model-based evaluation of intervention strategies. PLoS medicine 7, e1000324.

4. Sinden, R. (2010). A biologist’s perspective on malaria vaccine development. Human vaccines 6, 3-11.

5. WHO. (2001). Drug resistance in malaria. Peter B. Bloland Malaria Epidemiology Branch, Centers for Disease Control and Prevention, Chamblee, GA, United States of America. Pp.20

6. Alonso, P.L., Brown, G., Arevalo-Herrera, M., Binka, F., Chitnis, C., Collins, F., Doumbo, O.K., Greenwood, B., Hall, B.F., and Levine, M.M. (2011). A research agenda to underpin malaria eradication. PLoS medicine 8, e1000406.

7. Karunajeewa, H.A., and Mueller, I. (2016). How important is gametocyte clearance after malaria therapy? BMC medicine 14, 93.

8. Kuehn, A., and Pradel, G. (2010). The coming-out of malaria gametocytes. BioMed Research International 2010.

9. Sortica, V.A., Lindenau, J.D., Cunha, M.G., Ohnishi, M.D., Ventura, A.M.R., Ribeiro-dos-Santos, Â.K., Santos, S.E., Guimarães, L.S., and Hutz, M.H. (2016). The effect of SNPs in CYP450 in chloroquine/primaquine Plasmodium vivax malaria treatment. Pharmacogenomics 17, 1903-1911.

10. Koepfli, C., and Yan, G. (2018). Plasmodium Gametocytes in Field Studies: Do We Measure Commitment to Transmission or Detectability? Trends in parasitology.

11. Beavogui, A.H., Djimde, A.A., Gregson, A., Toure, A.M., Dao, A., Coulibaly, B., Ouologuem, D., Fofana, B., Sacko, A., and Tekete, M. (2010). Low infectivity of Plasmodium falciparum gametocytes to Anopheles gambiae following treatment with sulfadoxine–pyrimethamine in Mali. International journal for parasitology 40, 1213-1220.

12. Kone, A., Van de Vegte-Bolmer, M., Siebelink-Stoter, R., Van Gemert, G.-J., Dara, A., Niangaly, H., Luty, A., Doumbo, O.K., Sauerwein, R., and Djimde, A.A. (2010). Sulfadoxine–pyrimethamine impairs Plasmodium falciparum gametocyte infectivity and Anopheles mosquito survival. International journal for parasitology 40, 1221-1228.

13. Bejon, P., Andrews, L., Hunt-Cooke, A., Sanderson, F., Gilbert, S.C., and Hill, A.V. (2006). Thick blood film examination for Plasmodium falciparum malaria has reduced sensitivity and underestimates parasite density. Malaria Journal 5, 104.

14. Karl, S., Davis, T.M., and St-Pierre, T.G. (2009). A comparison of the sensitivities of detection of Plasmodium falciparum gametocytes by magnetic fractionation, thick blood film microscopy, and RT-PCR. Malaria journal 8, 98.

15. Young, J.A., Fivelman, Q.L., Blair, P.L., de la Vega, P., Le Roch, K.G., Zhou, Y., Carucci, D.J., Baker, D.A., and Winzeler, E.A. (2005). The Plasmodium falciparum sexual development transcriptome: a microarray analysis using ontology-based pattern identification. Molecular and biochemical parasitology 143, 67-79.

16. Silvestrini, F., Bozdech, Z., Lanfrancotti, A., Di Giulio, E., Bultrini, E., Picci, L., Pizzi, E., and Alano, P. (2005). Genome-wide identification of genes upregulated at the onset of gametocytogenesis in Plasmodium falciparum. Molecular and biochemical parasitology 143, 100-110.

17. Okell, L.C., Reiter, L.M., Ebbe, L.S., Baraka, V., Bisanzio, D., Watson, O.J., Bennett, A., Verity, R., Gething, P., Roper, C., et al. (2018). Emerging implications of policies on malaria treatment: genetic changes in the <em>Pfmdr-1</em> gene affecting susceptibility to artemether–lumefantrine and artesunate–amodiaquine in Africa. BMJ Global Health 3.

18. Falade, C., Makanga, M., Premji, Z., Ortmann, C.E., Stockmeyer, M., and de Palacios, P.I. (2005). Efficacy and safety of artemether-lumefantrine (Coartem) tablets (six-dose regimen) in African infants and children with acute, uncomplicated falciparum malaria. Transactions of the Royal Society of Tropical Medicine and Hygiene 99, 459-467.

19. Jima, D., Tesfaye, G., Medhin, A., Kebede, A., Argaw, D., and Babaniyi, O. (2005). Safety and efficacy of artemether-lumefantrine in the treatment of uncomplicated falciparum malaria in Ethiopia. East Afr Med J 82, 387-390.

20. Maiteki-Sebuguzi, C., Jagannathan, P., Yau, V.M., Clark, T.D., Njama-Meya, D., Nzarubara, B., Talisuna, A.O., Kamya, M.R., Rosenthal, P.J., Dorsey, G., et al. (2008). Safety and tolerability of combination antimalarial therapies for uncomplicated falciparum malaria in Ugandan children. Malaria journal 7, 106.

21. WHO. (2015). Guidelines for the Treatment of Malaria. In, W.H. Organization, ed. (Geneva.

22. Baird, J.K. (2005). Effectiveness of antimalarial drugs. The New England journal of medicine 352, 1565-1577.

23. FMoH. (2018). National Malaria Guideline. In, Disease, Prevention, andControl, eds. (Addis Ababa, Federal Ministry of Health, National Malaria Control and Elimination Program.

24. Hermsen, C.C., Telgt, D.S., Linders, E.H., Locht, L.A., Eling, W., and Mensink, E.J. (2001). Detection of Plasmodium falciparum malaria parasites in vivo by real-time quantitative PCR. Mol Biochem Parasitol 118.

25. Wampfler, R., Mwingira, F., Javati, S., Robinson, L., Betuela, I., Siba, P., Beck, H.P., Mueller, I., and Felger, I. (2013). Strategies for detection of Plasmodium species gametocytes. PLoS One 8, e76316.

26. Schneider, P., Schoone, G., Schallig, H., Verhage, D., Telgt, D., Eling, W., and Sauerwein, R. (2004). Quantification of Plasmodium falciparum gametocytes in differential stages of development by quantitative nucleic acid sequence-based amplification. Mol Biochem Parasitol 137, 35-41.

27. Koepfli, C., Robinson, L.J., Rarau, P., Salib, M., Sambale, N., Wampfler, R., Betuela, I., Nuitragool, W., Barry, A.E., Siba, P., et al. (2015). Blood-Stage Parasitaemia and Age Determine Plasmodium falciparum and P. vivax Gametocytaemia in Papua New Guinea. PLoS One 10, e0126747.

28. Stone, W., Sawa, P., Lanke, K., Rijpma, S., Oriango, R., Nyaurah, M., Osodo, P., Osoti, V., Mahamar, A., and Diawara, H. (2017). A Molecular Assay to Quantify Male and Female Plasmodium falciparum Gametocytes: Results From 2 Randomized Controlled Trials Using Primaquine for Gametocyte Clearance. The Journal of Infectious Diseases, jix237.

29. Tadesse, F.G., Lanke, K., Nebie, I., Schildkraut, J.A., Goncalves, B.P., Tiono, A.B., Sauerwein, R., Drakeley, C., Bousema, T., and Rijpma, S.R. (2017). Molecular Markers for Sensitive Detection of Plasmodium falciparum Asexual Stage Parasites and their Application in a Malaria Clinical Trial. Am J Trop Med Hyg 97, 188-198.

30. Poran, A., Notzel, C., Aly, O., Mencia-Trinchant, N., Harris, C.T., Guzman, M.L., Hassane, D.C., Elemento, O., and Kafsack, B.F.C. (2017). Single-cell RNA sequencing reveals a signature of sexual commitment in malaria parasites. Nature 551, 95-99.

31. Sinha, A., Hughes, K.R., Modrzynska, K.K., Otto, T.D., Pfander, C., Dickens, N.J., Religa, A.A., Bushell, E., Graham, A.L., Cameron, R., et al. (2014). A cascade of DNA-binding proteins for sexual commitment and development in Plasmodium. Nature 507, 253-257.

32. Kafsack, B.F., Rovira-Graells, N., Clark, T.G., Bancells, C., Crowley, V.M., Campino, S.G., Williams, A.E., Drought, L.G., Kwiatkowski, D.P., Baker, D.A., et al. (2014). A transcriptional switch underlies commitment to sexual development in malaria parasites. Nature 507, 248-252.

33. Blessborn, D., Romsing, S., Annerberg, A., Sundquist, D., Bjorkman, A., Lindegardh, N., and Bergqvist, Y. (2007). Development and validation of an automated solid-phase extraction and liquid chromatographic method for determination of lumefantrine in capillary blood on sampling paper. J Pharm Biomed Anal 45, 282-287.

34. Patchen, L.C., Mount, D.L., Schwartz, I.K., and Churchill, F.C. (1983). Analysis of filter-paper-absorbed, finger-stick blood samples for chloroquine and its major metabolite using high-performance liquid chromatography with fluorescence detection. Journal of chromatography 278, 81-89.

35. Tarning, J., and Lindegardh, N. (2008). Quantification of the antimalarial piperaquine in plasma. Transactions of the Royal Society of Tropical Medicine and Hygiene 102, 409-411.

# ANNEXES 4: Consent and Assent Forms

ENGLISH VERSION OF THE PARTICIPANT’S INFORMATION SHEET FOR THE AL plus single dose PQ ARMs (≥ 18 YEARS)

Flesch Kincaid Grade Level---- 8.8

**Study title: *Ethiopia in-vivo efficacy study 2019: Therapeutic efficacy of AL -Single low Dose PQ for the treatment of uncomplicated falciparum malaria and CQ -14 days PQ for uncomplicated vivax malaria.***

**Principal Investigators:** Dr Endalamaw Gadisa (Armauer Hansen Research Institute)

**Funder:** Ethiopian Federal Ministry of Health through SDG funding

INTRODUCTION

Hello! My name is _____________________________. I am a member of a malaria research team. We are doing a study to find out how efficacious the antimalarials used in Ethiopia are. The Armauer Hansen Research Institute is conducting the study in collaboration with FMOH. We are asking you/your child to join the study because you/your child are diagnosed with falciparum malaria. Your participation is voluntary. Please take your time to make your decision. Feel free to ask me any question(s).

WHAT IS THE PURPOSE OF THE STUDY?

The aim of this study is to learn how efficacious the AL-PQ to treat the malaria you/your child is diagnosed with. Your/your child’s medication, AL-PQ is as per the national malaria guideline.

WHY AM I ASKED TO BE/MY CHILD BE PART OF THIS STUDY?

You/your child are diagnosed to have falciparum malaria.

HOW MANY PEOPLE WILL TAKE PART IN THIS STUDY?

Eighty eight people who are diagnosed to have falciparum malaria will take part in this study.

WHAT WILL HAPPEN IF I/MY CHILD TAKE PART IN THIS STUDY?

If you agree, we will enroll you/your child into the study. We will ask you some questions, take your/your child’s weight (arm circumference) and you will have physical examination. In addition, we request you to return to the clinic for additional 9 days; we will give you an appointment Card so that you know what days to come. At each visit, we will request about 1 mL of blood. We will use this blood to test for malaria, Hg level and to prepare blood spots on a filter paper. In addition, we will request all females of childbearing age to get checked for pregnancy, volunteers who tested positive will be excluded and handled as per the national guideline. The blood spots will be sent to a reference laboratory in Addis Ababa. There, we will check for malaria and marker of drug resistance. We will tell you the result of the smear and Hg, but as are not directly relevant for your treatment you will not receive the results of tests done at the reference laboratory.

HOW LONG WILL I/MY CHILD BE IN THE STUDY?

The duration of your/your child’s participation in the study will be 42 days.

CAN I WITHDRAW MY CONSENT FOR THE STUDY?

Participation is fully on voluntary bases: You/your child may drop out at any time. Doing so, will not affect the care that you/ your child receive for any case in any way.

WHAT RISK CAN I EXPECT FROM BEING IN THE STUDY?

*Risks of Taking Drugs:* We will use drugs recommended to treat malaria. The drugs can cause an upset stomach, vomiting, diarrhea, headache, dizziness, mild skin rash, and itching. These are mostly mild. Rarely, more severe side effects can occur. If you have severe unhealthy feelings or darkening of your/your child’s urine, stop taking the drug and return to the health facility. The study will pay for any additional treatment costs.

*Risk of Blood Collection:* it might cause minor pain, bleeding, and bruising. Very rarely, fainting or secondary infection might occur. To minimize risk, only health staff trained on the specific protocol will take samples following accepted aseptic procedures.

ARE THERE BENEFITS TO TAKING PART IN THE STUDY?

You will be closely followed for the next 42 days to see how efficacious the drugs are and taken care of if deemed necessary. Also this study will help the Ministry of Health to better understand how efficacious the drugs are to treat falciparum malaria.

WHAT ARE THE COSTS OF TAKING PART IN THIS STUDY?

There will be no monitory costs to you/your child to participate in the study.

WILL I BE PAID FOR TAKING PART IN THIS STUDY?

You/your child will not be paid for taking part in this study; we will give you 100 Birr for transportation for the scheduled visits.

WHAT ARE MY RIGHTS IF I/MY CHILD TAKE PART IN THIS STUDY?

Taking part in this study is entirely on voluntary bases. If you decide to take part or your child take part in the study, and then later want to leave, you/your child can stop at any time. No matter what decision you make, there will be no penalty to you/your child in any way. You/your child have the right to withhold information, decline to cooperate in the study and refuse provision of specimens.

HOW WILL MY/MY CHILD’S *SPECIMENS AND* INFORMATION BE USED?

Researchers will use your/your child’s blood samples and information for this study. Once the study is done using the blood samples and information, we may share de-identified information with other researchers/program people so they can use them for programmatic or research studies in the future. We will not share names or any other personal information that would let the researchers know who you/your child are. Please let the researcher know if you do not want your/your child’s samples be used for future similar use. If you agree, we will not ask you for additional permission to share this de-identified information.

WILL INFORMATION FROM THE RECORDS BE KEPT PRIVATE?

The information about you/your child will be kept confidential to the extent allowed by law. Your/your child’s name will not be used on labels or in any report resulting from this study. Any information obtained will be kept locked and password protected. By signing this document, you grant permission for information about you/your child to be made available to the investigator, staff members, and other medical staff who may be evaluating the study.

WHO CAN ANSWER MY QUESTIONS ABOUT THE STUDY?

If you have any questions, please speak with one of the study staff members. If you have any questions about this study later, please contact the principal investigators; Dr. Endalamaw Gadisa at 0911868827. If you wish to ask questions about the study or your rights as a research participant, please call the Chairperson of the AHRI/ALERT ethics review committee at 0118962183.

**CONSENT**

If you wish to participate, you should sign below.

**Participant Consent Signature Form for participation in the AL plus single dose PQ Arms**

Statement of Consent for participation (signature or thumbprint required)

The above has been read to me, and I agree to take part in the survey.

Signature: ____________________________ Date: ________________

Thumb print:

Participant’s name: ____________________

For persons who cannot sign

The above consent was read and the person agreed to take part.

Signature: ____________________________ Date: ________________

Witness’s name: _______________________

Study team interviewer

Name: ___________________________________ Date: __________________

Signature: ________________________________

**NOTE:**  This consent form with original signatures must be retained on file by the principal investigator. A copy must be given to the participant. If the participant refuses to take her copy of the consent form with him/her, please ask him/her to sign and date the decline statement below.

I have been offered a copy of this consent form and decline to take it.

Participant’s signature ________________________________ Date ________________

**Parent/guardian Consent Form for minors, in the AL plus single dose PQ Arms**

Statement of Consent for participation for child **< 18 years of age**:

The above has been read to me.

□ YES, I agree for my child to take part in the study.

□ NO, I do not agree for my child to take part in the study.

Participant’s name: ____________________ Parent/Guardian’s name: _________________

**For persons who cannot sign**

□ The above consent was read and the person agreed for their child, to take part.

□ The above consent was read and the person did NOT agree for their child, to take part.

Participant’s name: ____________________ Parent/Guardian’sname: ________________

Signature: ____________________________ Date: ________________

Witness’s name: _______________________

I have explained the purpose of this study to the volunteer. To the best of my knowledge, he/she understands the purpose, procedures, risks and benefits of this study.

Study team interviewer

Name: _______________________________Signature: ________________ Date: ________

**NOTE:**  This consent form with original signatures must be retained on file by the principal investigator. A copy must be given to the participant. If the participant refuses to take her copy of the consent form with him/her, please ask him/her to sign and date the decline statement below.

I have been offered a copy of this consent form and decline to take it.

Participant’s signature ________________________________ Date ________________

**ASSENT FORM FOR MATURE MINORS (12–17 YEARS) PARTICIPATES IN THE AL PLUS PQ ARM**

Flesch Kincaid Grade Level----5.0

**Study title: *Ethiopia in-vivo efficacy study 2019: Therapeutic efficacy of AL -Single low Dose PQ for the treatment of uncomplicated falciparum malaria and CQ -14 days PQ for uncomplicated vivax malaria.***

**Principal Investigators:** Dr Endalamaw Gadisa (AHRI)

**Funder:** Ethiopian Federal Ministry of Health through SDG fund

INTRODUCTION

Hi! My name is _____________________________. I am a part of a study team. We are doing a study know how well the malarial used to treat your type of case are efficacious. This study will be done by AHRI in collaboration with FMOH. You are confirmed to have falciparum malaria. So, I would like to invite you to join this study. Please take your time to decide, feel free to ask me any questions; as well we will wait until your parents give permission for you to participate in the study. This study is very important to help our government to learn how effective the malarial in use are or prepare in case not.

WHAT IS THE PURPOSE OF THE STUDY?

The study team needs to learn how efficacious are the antimalarials used to treat as per the guideline and assess alternative to make informed decision if deemed.

WHY AM I BEING ASKED TO BE A PART OF THIS STUDY?

You have falciparum malaria; so you are eligible for testing and treatment.

HOW MANY PEOPLE WILL TAKE PART IN THIS STUDY?

About 88 people who are confirmed to have your type of malaria will take part in this study.

WHAT WILL HAPPEN IF I TAKE PART IN THIS RESEARCH STUDY?

If you agree to participate in this study, we will ask you some questions. About 1 mL of blood will be taken to test for species malaria, stages and anemia. In addition, we request you to return to the clinic for additional 9 days; we will give you an appointment Card so that you know what days to come. At each visit, we will request about 1 mL of blood. We will provide you/your child treatment as per the national guideline: AL and single dose of 0.25 PQ/Kg.

HOW LONG WILL I BE IN THE STUDY?

You will be in the study for 42 days. If you do not appear on appointment we will make a reminder call or someone from the project could visit you.

CAN I STOP BEING IN THE STUDY?

Yes, you can decide to stop at any time for any reason.

WHAT RISK CAN I EXPECT FROM BEING IN THE STUDY?

***Risks of taking drugs***: You will get antimalarials proved to be safe to treat your type of malaria.

***Risk of finger prick***: The blood collection has minor pain and can rarely bleed or gets infected. We will make sure a trained health professional do the collection. We will use new needles each time.

ARE THERE BENEFITS TO TAKING PART IN THE STUDY?

You will be closely followed for the next 42 days to see how efficacious the drugs are and taken care of if deemed necessary. Also this study will help the Ministry of Health to better understand how efficacious the drugs are to treat malaria.

WHAT ARE THE COSTS OF TAKING PART IN THIS STUDY?

There are no monitory costs.

WILL I BE PAID FOR TAKING PART IN THIS STUDY?

You will not be paid for taking part in this study. We will provide 100 birr as a compensation for transport on scheduled visits; we will provide an appointment card.

WHAT ARE MY RIGHTS IF I TAKE PART IN THIS STUDY?

Taking part in this study is entirely your choice. If you decide to take part in the study, and you may want to leave later, you can stop at any time. No matter what decision you make, nothing bad will happen to you.

If you agree, we would like you to join the study.

**STATEMENT OF ASSENT**

If you say yes, to take part in this research study we will put a check mark alongside your name to confirm the same.

Date Name

Date Name of Parent/Guardian Signature or finger print

Date Name of Person Obtaining Assent Signature

ENGLISH VERSION OF THE PARTICIPANT’S INFORMATION SHEET FOR THE CQ plus 14 days PQ ARMs (≥ 18 YEARS)

Flesch Kincaid Grade Level---- 8.8

**Study title: *Ethiopia in-vivo efficacy study 2019: Therapeutic efficacy of AL -Single Dose PQ for the treatment of uncomplicated falciparum malaria and CQ -14 days PQ for uncomplicated vivax malaria.***

**Principal Investigators:** Dr. Endalamaw Gadisa (Armauer Hansen Research Institute)

**Funder:** Ethiopian Federal Ministry of Health through SDG fund

INTRODUCTION

Hello! My name is _____________________________. I am a member of a malaria research team. We are doing a study to find out how efficacious the antimalarials used in Ethiopia are. The Armauer Hansen Research Institute is conducting the study in collaboration with FMOH. We are asking you you/your child to join the study because you/your child are diagnosed with falciparum malaria. Your participation is voluntary. Please take your time to make your decision. Feel free to ask me any question(s).

WHAT IS THE PURPOSE OF THE STUDY?

The aim of this study is to learn how best to treat vivax malaria with CQ-PQ, the type you/your child are diagnosed to have. Your/your child’s medication, CQ-PQ was decided by chance.

WHY AM I ASKED TO BE/MY CHILD BE PART OF THIS STUDY?

You/your child are diagnosed to have vivax malaria.

HOW MANY PEOPLE WILL TAKE PART IN THIS STUDY?

Eighty eight people who are diagnosed to have vivax malaria will take part in this study.

WHAT WILL HAPPEN IF I TAKE PART IN THIS STUDY?

If you agree to participate, we will enroll you/your child into the study. We will ask you some questions, take your/your child’s weight (arm circumference) and you will have physical examination. In addition, we request you to return to the clinic for additional 9 days; we will give you an appointment Card so that you know what days to come. At each visit, we will request about 1 mL of blood. We will use this blood to test for malaria, Hg level and to prepare blood spots on a filter paper. In addition, we will request all females of childbearing age to get checked for pregnancy, volunteers who tested positive will be excluded and handled as per the national guideline. The blood spots will be sent to a reference laboratory in Addis Ababa. There, we will check for malaria and marker of drug resistance. We will tell you the result of the smear/RDT and Hg, but as are not directly relevant for your treatment you will not receive the results of tests done at the reference laboratory.

We will provide you/your child CQ; plus 14 days of 0.25 PQ/Kg. You/your child will need to visit the health post as per the schedule card. We will also provide and train you a urine color monitoring chart.

HOW LONG WILL I/MY CHILD BE IN THE STUDY?

The duration of your/your child’s participation in the study will be 42 days.

CAN I WITHDRAW MY CONSENT FOR THE STUDY?

Participation is fully on voluntary bases: You/your child may drop out at any time. It will not affect the care that you/ your child receive for any case in any way.

WHAT RISK CAN I EXPECT FROM BEING IN THE STUDY?

*Risks of Taking Drugs:* We will use drugs recommended to treat malaria. The drugs can cause an upset stomach, vomiting, diarrhea, headache, dizziness, mild skin rash, and itching. These are mostly mild. Rarely, more severe side effects can occur. If you have severe unhealthy feelings or darkening of your/your child’s urine, stop taking the drug and return to the health facility. The study will pay for any additional treatment costs.

*Risk of Blood Collection:* it might cause minor pain, bleeding, and bruising. Very rarely, fainting or secondary infection might occur. To minimize risk, only health staff trained on the specific protocol will take samples following accepted aseptic procedures.

ARE THERE BENEFITS TO TAKING PART IN THE STUDY?

You will be closely followed for the next 42 days to see how efficacious the drugs are and taken care of if deemed necessary. Also this study will help the Ministry of Health to better understand how efficacious the drugs are to treat malaria.

WHAT ARE THE COSTS OF TAKING PART IN THIS STUDY?

There will be no monitory costs to you/your child to participate in the study.

WILL I BE PAID FOR TAKING PART IN THIS STUDY?

You/your child will not be paid for taking part in this study; we will give you 100 Birr for transportation for the scheduled visits.

WHAT ARE MY RIGHTS IF I/MY CHILD TAKE PART IN THIS STUDY?

Taking part in this study is entirely on voluntary bases. If you decide to take part or your child take part in the study, and then later want to leave, you/your child can stop at any time. No matter what decision you make, there will be no penalty to you/your child in any way. You/your child have the right to withhold information, decline to cooperate in the study and refuse provision of specimens.

HOW WILL MY/MY CHILD’S *SPECIMENS AND* INFORMATION BE USED?

Researchers will use your/your child’s blood samples and information for this study. Once the study is done using the blood samples and information, we may share de-identified information with other researchers/program people so they can use them for programmatic or research studies in the future. We will not share names or any other personal information that would let the researchers know who you/your child are. Please let the researcher know if you do not want your/your child samples be used for future similar use. If you agree, we will not ask you for additional permission to share this de-identified information.

WILL INFORMATION FROM THE RECORDS BE KEPT PRIVATE?

The information about you/your child will be kept confidential to the extent allowed by law. Your/your child’s name will not be used on labels or in any report resulting from this study. Any information obtained will be kept locked and password protected. By signing this document, you grant permission for information about you/your child to be made available to the investigator, staff members, and other medical staff who may be evaluating the study.

WHO CAN ANSWER MY QUESTIONS ABOUT THE STUDY?

If you have any questions, please speak with one of the study staff members. If you have any questions about this study later, please contact the principal investigators Dr. Endalamaw Gadisa at 0911868827. If you wish to ask questions about the study or your rights as a research participant, please call the Chairperson of the AHRI/ALERT ethics review committee at 0118962183.

**CONSENT**

If you wish to participate, you should sign below.

**Participant Consent Signature Form for participation in the CQ plus 14 days PQ Arms**

Statement of Consent for participation (signature or thumbprint required)

The above has been read to me, and I agree to take part in the survey.

Signature: ____________________________ Date: ________________

Thumb print:

Participant’s name: ____________________

For persons who cannot sign

The above consent was read and the person agreed to take part.

Signature: ____________________________ Date: ________________

Witness’s name: _______________________

For persons who cannot sign

The above consent was read and the person agreed to allow their malaria tests to be saved for future testing.

Signature: ____________________________ Date: ________________

Witness’s name: ____________________

Study team interviewer

Name: ___________________________________ Date: __________________

Signature: ________________________________

**NOTE:**  This consent form with original signatures must be retained on file by the principal investigator. A copy must be given to the participant. If the participant refuses to take her copy of the consent form with him/her, please ask him/her to sign and date the decline statement below.

I have been offered a copy of this consent form and decline to take it.

Participant’s signature ________________________________ Date ________________

**Parent/guardian Consent Form for minors, in the CQ plus 14 days PQ Arms**

Statement of Consent for participation for child **< 18 years of age**:

The above has been read to me.

□ YES, I agree for my child to take part in the study.

□ NO, I do not agree for my child to take part in the study.

Signature: ____________________________ Date: ________________

Thumb print:

Participant’s name: ____________________ Parent/Guardian’sname: _________________

**For persons who cannot sign**

□ The above consent was read and the person agreed for their child, to take part.

□ The above consent was read and the person did NOT agree for their child, to take part.

Participant’s name: ____________________ Parent/Guardian’sname: _____________

Signature: ____________________________ Date: ________________

Witness’s name: _______________________

I have explained the purpose of this study to the volunteer. To the best of my knowledge, he/she understands the purpose, procedures, risks and benefits of this study.

Study team interviewer

Name: _______________________________ Signature: ________________ Date: ___________

**NOTE:**  This consent form with original signatures must be retained on file by the principal investigator. A copy must be given to the participant. If the participant refuses to take her copy of the consent form with him/her, please ask him/her to sign and date the decline statement below.

I have been offered a copy of this consent form and decline to take it.

Participant’s signature ________________________________ Date ________________

**ASSENT FORM FOR MATURE MINORS (12–17 YEARS) PARTICIPATES IN THE CQ PLUS PQ ARM**

Flesch Kincaid Grade Level----5.0

**Study title: *Ethiopia in-vivo efficacy study 2019: Therapeutic efficacy of AL -Single Dose PQ for the treatment of uncomplicated falciparum malaria and CQ -14 days PQ for uncomplicated vivax malaria.***

**Principal Investigators:** Dr. Endalamaw Gadisa (AHRI)

**Funder:** Ethiopian Federal Ministry of Health through SDG fund

INTRODUCTION

Hi! My name is _____________________________. I am a part of a study team. We are doing a study know how well the malarial used to treat your type of case are efficacious. This study will be done by AHRI in collaboration with FMOH. You are confirmed to have falciparum malaria. So, I would like to invite you to join this study. Please take your time to decide, feel free to ask me any questions; as well we will wait until your parents give permission for you to participate in the study. This study is very important to help our government to learn how effective the malarial in use are or prepare in case not.

WHAT IS THE PURPOSE OF THE STUDY?

The study team needs to learn how efficacious are the antimalarials used to treat as per the guideline and assess alternative to make informed decision if deemed.

WHY AM I BEING ASKED TO BE A PART OF THIS STUDY?

You have vivax malaria; so you are eligible for testing and treatment.

HOW MANY PEOPLE WILL TAKE PART IN THIS STUDY?

About 350 people who are confirmed to have your type of malaria will take part in this study.

WHAT WILL HAPPEN IF I TAKE PART IN THIS RESEARCH STUDY?

If you agree to participate in this study, we will ask you some questions, about 1 mL of blood will be taken to test for species malaria, stages and anemia. In addition, we request you to return to the clinic for additional 9 days; we will give you an appointment Card so that you know what days to come. At each visit, we will request about 1 mL of blood.

We will provide you/your child the CQ–plus 14 days of 0.25 PQ/Kg. You/your child will need to visit the health post as per the schedule card to be provided.

HOW LONG WILL I BE IN THE STUDY?

You will be in the study for 42 days. If you do not appear on appointment we will make a reminder call or someone from the project could visit you.

CAN I STOP BEING IN THE STUDY?

Yes, you can decide to stop at any time for any reason.

WHAT RISK CAN I EXPECT FROM BEING IN THE STUDY?

***Risks of taking drugs***: You will get antimalarials proved to be safe to treat your type of malaria. In case you have darkening of urine and/or any uncomfortable unusual scenarios stop taking the medication and come back to the health facility.

***Risk of finger prick***: The blood collection has minor pain and can rarely bleed or gets infected. We will make sure a trained health professional do the collection. We will use new needles each time.

ARE THERE BENEFITS TO TAKING PART IN THE STUDY?

You will be closely followed for the next 42 days to see how efficacious the drugs are and taken care of if deemed necessary. Also this study will help the Ministry of Health to better understand how efficacious the drugs are to treat malaria.

WHAT ARE THE COSTS OF TAKING PART IN THIS STUDY?

There are no monitory costs.

WILL I BE PAID FOR TAKING PART IN THIS STUDY?

You will not be paid for taking part in this study. We will provide 100 birr as a compensation for transport on scheduled visits; we will provide an appointment card.

WHAT ARE MY RIGHTS IF I TAKE PART IN THIS STUDY?

Taking part in this study is entirely your choice. If you decide to take part in the study, and you may want to leave later, you can stop at any time. No matter what decision you make, nothing bad will happen to you.

If you agree, we would like you to join the study.

**STATEMENT OF ASSENT**

If you say yes, to take part in this research study we will put a check mark alongside your name to confirm the same.

Date Name

Date Name of Parent/Guardian Signature or finger print

Date Name of Person Obtaining Assent Signature

# Appendix 4: Amharic version of information sheet II

**የጥናት መረጃ ቅጽ**

**ጥናቱ ተሳታፊ የመረጃ ፎርም ዕድሜያቸው ከ ≥18 ዓመት በላይ ለሆኑ ተሳታፊዎች**

**ለተሳታፊዎች መረጃ**

**የጥናቱ ርዕስ፡** የፈልሲፓረም ወባ መድሃኒቶች ፈዋሽነት ማረጋገጫ ጥናት

ዋና ተመራማሪ፡ ዶ/ር እንዳለማው ጋዲሳ(ከአርማወር ሀንሰን የምርምር ተቋም)

የጥናቱ በጀት ደጋፊ፡ የኢትዮጲያ ጤና ጥበቃ ሚኒስተር

**መግቢያ**

እንደምን አደራችሁ/ዋላችሁ፡ስሜ------------------------------------እኔ በአርማወር ሀንሰን የምርምር ተቀም በወባ እና ትኩረት የሚሹ የሀሩር በሽታዎች የጥናት ቡድን አባል ነኝ፡፡ እኛ አሁን የጥምረት የጸረ-ወባ መዳኒት የፈዋሽኝነት አቅም በኢትዮጲያ ማጥናት ይሆናል፡፡ይህን ጥናት በፌደራል ጤና ጥበቃ ሚኒስተር ይደገፋል፡፡ ስለሆነም እርሶ ወይም ልጅዎ በጥናቱ እንዲሳተፉ እንጠይቃለን ምክንያቱም በደሞ ውስጥ የወባ አምጪ ተዋሲያን ሰስለተገኘ ነው፤ጥናቱ በፍጹም ፈቃደኝነት ላይ የተመሰረተ ነው፡፡

**የጥናቱ ዓላማ**

ባለፉት አስር ዓመታት ተጨባጭ የሆነ የወባ ስርጭትና በወባ ምክንያት የሚከሰት ሞት መቀነስ ታይቷል። ይሁን እንጂ ይህ ስኬት ቀጣይ እንደሆነና የወባ በሽታ ከኢትዮጵያ ለማስወገድ አንዱ ፈታኝ ሁኔታ የወባ በሽታ አምጪ ተዋሲያን አሁን ያሉት ጸረ-ወባ መጋኒቶች የማዳን አቅም እየተዳከመ መምጣቱን ተከትሎ የማዳን አቅም ከፍ ለማድረግ እነዲያስችል አሁን ካሉት የመጀመሪያ ደረጃ ፀረ ወባ መድሐኒቶች ጋር ተጨማሪ የፀረ ወባ መድሐኒቶች በጥምረት በመጠቀም የማዳን አቅም እና የጎኑዮሽ ጉዳት ማጥናት ይሆና፡፡ ስለሆነም ይህ ጥናት ዓላማ አርተሜሲኒን ሉፋትሪን ከ ፕሪማከን ጋር ላልተወሳሰበ ፕላስሞዲየም ፈልስፋረምን የማዳን አቅም መፈተሸ ይሆናል፡፡

በመሆኑም ይህ ጥናት ሃገሪቱ ላቀደችው ወባን ፈጽሞ የማጥፋት አላማ ጋር ተያይዞ የፀረ ወባ መዳኒቶች የማዳን አቅም በየጊዜው መፈተሽ አስፈላጊነቱን ማጥናት እና ጥሩ እና ወሳኝ ግብአት እንዲሆን ማድረግ ይሆናል። ከዚህም የሚገኘው መረጃ ከሃገራችን ኢትዮጵያም አልፎ ተመሳሳይ ሁኔታ ላይ ላሉ ሌሎች ሃገራትም ጠቀሜታው የጎላ ይሆናል። ማንኛውም ጥናት ተያያዥ የሆነ የምቾት መጓደል ሊያመጣ ይችላል። ከዚህም ተያይዞ የሚቻለንን ያህል ሊኖሩ የሚችሉትን የምቾት መጓደሎች ለመቀነስ ጥረት አድርገናል። በማንኛውም ደረጃ አለም አቀፍ እና እንዲሁም በሃገራችን የተደነገጉ ህግጋትን ጠብቀን የህክምና ስነ-ምግባር በሚፈቅደው መሰረት እንሰራለን።

ስለሆነም እርሶ ወይም ልጅዎ በዚህ ጥናት ለመሳተፍ ፈቃደዎን እነዲገልጹልን እንጠይቅዎታለን። ከእርሶ/ከልጅዎ የሚሰበሰበው የደም ናሙና ወደፊት ከወባ መስፋፋት ጋር ብቻ ለተያያዙ ጥናቶች ጥቅም ላይ ይውላል። በናሙናዎቹ ላይ ተጨማሪ ጥናት በሚያስፈልግበት ሁኔታ ከሚመለከተው አካል ፍቃድ እንጠይቃለን፣ እንደ ስምና አድራሻ ያሉ ግለሰባዊ መረጃዎች ከመረጃው ይነጠላሉ፤ የጥናት የስነምግባር ይሁንታም ይጠየቅባቸዋል።

**ጥናቱን ለመሳተፍ ቅድመ ሁኔታ**

እርሶ ወይም ልጅዎ ይህን ጥናት ለመሳተፍ በመጀመሪያ በእርሶ ወይም በልጅዎ ደም ውስጥ የወባ አምጪ ተዋሲያሰን መገኘት አለበት፡፡

**ለጥናቱ የሚያስፈልጉ ተሳታፊዎች**

በአጠቃላይ 88 የፋልሲፓረም የወባ ተዋሲያን በደማቸው የተገኘባቸው ሰዎች በጥናቱ ይሳተፋሉ፡፡

**የጥናቱ ሂደት**

ውድ የጥናቱ ተሳታፊ፡ይህን የመረጃ ቅጽ ለመሙላት ወደ 20 ደቂቃ ይፈጃል። ላሳዩት ትግስት እናእርሶ/ ልጅዎ በጥናቱ ለመሳተፉ በመፍቀድዎ አስቀድመን ልናመሰግንዎት እንወዳልን።

እርሶ/ልጅዎ በጥናቱ ለመሳተፍ የእርስዎን ፍቃደኝነት እንጠይቃለን። ለዚህም እንዲረዳን የተለያዩ ምርመራዎችን ለማድረግ የሚረዳ 5 ሚሊ ሌትር የደም ናሙና በጥናቱ መጀመሪያ ላይ እንዲሰጡን ይጠየቃሉ፡፡ ከዚህ በኋላ ለተከታታይ ቀናቶች ከጣት ላይ(በ 0, 2, 3, 7, 10, 14, 21, 28, 42 የደም ናሙና እርሶ/ልጅዎ እንዲሰጡ እንጠይቃለን:: በጥናቱ ወቅት የምንወስደው የደም መጠን ከ 1ሚሊ እና 5 ሚሊ መካከል ይገኛል፡፡ ይህ የደም መጠን በ 2 ወር ጊዜ ውስጥ በመከፋፈል የሚወሰድ ይሆናል፡፡ ለዚህም ይረዳ ዘንድ በቅድሚያ በመረዳት ላይ የተመረኮዘ ተሳትፎ እንዲያደርጉ በጥናቱ ለመሳተፍ ፍቃድዎን እንዲገልጹልን እንጠይቃለን፡፡

ሁሉም ሰው የሰውነት ሙቀት መጨመር እና የወባ በሽታ ተመርምሮ ከተገኘበት ወዲያውኑ ህክምና ያገኛል፡፡ ከዚያም ለተከታታይ ቀናት (0, 1 2, 3, 7, 10, 14, 21, 28, 42) ክትትል ይደረጋል፡፡

**በፈቃደኝነት ላይ የተመሰረተ ተሳትፎ**

እርሶ/ልጅዎ በጥናቱ እንዳይሳተፍ ወይም እንዲያቋርጥ መወሰንዎ ልጅዎም ሆነ እርሶ ሊያገኙ የሚችሉት የጤና አገልግሎት ላይ ምንም ተፅእኖ አይኖረውም። እርሶ/ልጅዎ ጥናቱን ማረጥ ከፈለጋችሁ በማንኛውም ጊዜ ማቋረጥ ይችላሉ። እርሶ/ልጅዎ በጥናቱ እንዳይሳተፍ ቢወስኑም እንኳን ተገቢውን አገልግሎት ያገኛሉ።

**የምቾት መጓደልና ተጋላጭለት**

ከእርሶ/ልጅዎ የደም ናሙና በሚወሰድበት ጊዜ የተወሰነ የምቾት መጓደል ሊኖር ይችላል፤ ደም በተወሰደበትም ቦታ የተወሰነ የመቅላት ወይም መድማት ሊኖር ይችላል። ይህ ክስተት ግን ጎጂ እንዳልሆነ ይታሰባል። ናሙናዎችን ለመውሰድ ከጀርም የጸዱ መገልገያዎችን እንጠቀማለን፤ ከናሙና መውሰድ ጋር በተገናኘ ሊከሰት የሚችልን መጠነኛ ቁስለት ቢኖር እንኳን በተገቢው እናክማለን። የሚወሰደው የደም መጠን በጤና ላይ ተጽኖ ለማስከትል በጣም ትንሽ ነው፤ የተወሰደው ደምም በሰውነት በቶሎ ይተካል።

**ጠቀሜታ**

እርሶ/ልጅዎ በጥናቱ ጊዜ ከወባ ጋር የተያያዘ ነፃ የህክምና አገልግሎት ታገኛለችሁ። የተለየ አትኩሮት የሚፈልጉ ሁኔታዎች ከተከሰቱ በጤና ተቋም ህክምና እንዲያገኙ ይደረጋል። እርሶም ሆኑ ልጅዎ በጥናቱ በመሳተፋችሁ እና ላጠፉት ጊዜ ወይም ለትራንስፖርት እንደማካካሻ የምታገኙት 100 ብር ክፍያ ይኖራል፡፡

**በጥናቱ የመሳተፍ ነጻነት**

ጥናቱ በሙሉ ፈቃደኝነት ላይ የተመሰረተ ነው፡፡እርሶ ወይም ልጅዎ በጥናቱ ለመሳተፍ ከወሰኑ መሳተፍ ይችላሉ፡ነገር ግን እርሶ ወይም ልጅዎ በማንኛውም ጊዜ ከጥናቱ መውጣት ከፈለጉ መውጣት ይችላሉ፡፡ለዚህም በእርሶም ሆነ በልጅዎ ላይ የሚደርስ ቅጣት የለም፡፡

**ናሙናዎችን ጥናቱ ጊዜ እስከሚያልቅ ስለማስቀመጥ**

ከእርሶና ከልጅዎ የሚገኘውን ናሙና የጥናቱ ጊዜ እስከሚያልቅ ማስቀመጥ እንድንችል ፈቃድዎትን እንጠይቃለን። ናሙናዎች የሚቀመጡት የጥናቱ ጊዜ እስኪጠናቀቅ ጊዜ በቻ ይሆናል፡፡ ናሙናዎችን የምንጠቀማቸው የወባ ጸረ-መዳኒቶች ላይ ያላቸውን አቅም ከመፈተሸ ጋር በተዛመደ ጥናት፤ የሚረዱ መረጃዎችን ለማግኘት ብቻ ይሆናል። ተጨማሪ ጥናት አስፈላጊ ከሆነ የስነምግባር ኮሚቴ ይሁንታን የሚጠየቅበት ይሆናል።

**ሚስጥርን ስለመጠበቅ**

ከእርሶ ወይም ከልጅዎ መሳትፍ የሚገኙት መረጃዎች ለዚህ ጥናት ጠቀሜታ ብቻ ይውላሉ። የርሶም ሆነ የልጅዎ ስም ለናሙና መለያነት ወይም በማንኛውም የዚህ ጥናት ውጤት ሪፖርት ላይ አንጠቀምም። በጥናቱ መጀመሪያ ለተሳታፊዎች የመለያ የሚስጥር ቁጥር ይሰጣል፤ ይህም ለናሙናዎችና በጥናቱ በጥቅም ላይ ለሚውሉ ቅጾች መለያነት ይጠቅማል። ማንኛውም ከዚህ ጥናት በተዛመደ የሚገኝ መረጃ በሚስጥር ይያዛል፤ መረጃዎችም በቁልፍ ተቆልፎባቸው ይቀመጣሉ። የእርስዎንም ሆነ የልጅዎን ስም ከጥናቱ የሚስጥር ቁጥር ጋር የሚያገናኘውን መረጃ የጥናቱ ዋና ተመራማሪዎች ብቻ ናቸው ማየት የሚችሉት።

**ጥያቄዎችን የመጠየቅና ከጥናቱ አቋርጦ የመውጣት ነጻነት**

ከጥናቱ ጋር በተያያዘ ጥያቄ ካላችሁ፤ የጥናቱን ዋና ተመራማሪ ዶ/ር እንዳላማው ጋዲሳን ለመጠየቅ አያመንቱ። አርማወር ሀንሰን የምርምር ተቋም, ፖሰት ሳጥን ቁጥር 1005, አዲስ አበባ, ኢትዮጵያ ስልክ ቁጥር፦0911868827)። ስለጥናቱ እና ከጥናቱ ጋር ያልተገናኘ ገለልተኛ ወገንን ማማከር ካስፈለጋችሁ እንደጥናቱ ተሳታፊነታችሁ የአርማዉር ሃንሰን ምርምር ተቋም/ የአለርት የስነ-ምግባር ኮሚቴ ጸሃፊን በተመለከተው አድራሻ ማግኘት ይችላሉ (0118-962183)።

**የጥናቱ ተሳታፊ የስምምነት መግለጫ ቅጽ ዕድሜያቸው ከ 18 ዓመት በላይ ለሆኑ ተሳታፊዎች**

እኔ ___________________________________ባለኝ ሙሉ ሃላፊነት ጥናት ውስጥ እንድሳተፍ ተስማምቻለሁ። በጥነቱ ተሳታፊ የሆንኩት በገዛ ፍቃዴ ነዉ፡፡ ያለምንም ቅጣት በማንኛውም የጥናቱ ወቅት ፈቃዴን ማንሳትና ከጥናቱ መገለል እንደምችል ተረድቻለሁ። ሰለሆነም በጥናቱ ለመሳተፍ ፍቃደኝነቴን በፊርማየ አረጋግጣለሁ፡፡ የስምምነት መግለጫ ቅጹ ግልባጭ ይሰጠወታል፡፡

ፊርማ|አሻራ፦ ____________ ቀን፦ ________________

የተሳታፊው ሰም፦ ___________________________

መፈረም ለማይችሉ፤

ተሳታፊዉ የስምምነት መግለጫ ቅጹ ተነቦለት በጥናቱ ለመሳተፍ ፍቃደኝነቱን መግለጹን እመሰክራለሁ፡፡

የምስክር ስም፦_______________________ፊርማ|አሻራ፦____________ ቀን፦ ___________

የተሳታፊው ሰም፦ ___________________________

መፈረም ለማይችሉ፤

ተሳታፊዉ የስምምነት መግለጫ ቅጹ ተነቦለት በጥናቱ ለመሳተፍ ፍቃደኝነቱን መግለጹን እመሰክራለሁ፡፡

የምስክር ስም፦_______________________ፊርማ|አሻራ፦____________ ቀን፦ ___________

**ለወላጅ/አሳዳጊ የስምምነት መግለጫ ቅጽ ዕድሜያቸው <18 ዓመት**

**የጥናቱ ርእስ፡-** የፈለሲፓረም ወባ መድሃኒቶች ፈዋሽነት ማረጋገጫ ጥናት

እኔ ወላጅ/አሳዳጊ, በጥናቱ ልጄ እንዲሳተፍ የተሳታፊነት ማሳወቂያ ቅፁን ከመፈረሜ አስቀድሞ ሥለጥናቱ አካሄድ እና ከዚህ ቀጥሎ የተዘረዘሩትን የጥናቱ ተሳታፊ መብቶች ግልፅ በሆነ መልኩ ከመረጃ ቅፁ አንብቤ ተረድቻለሁ /ተነቦልኝ በግልፅ ተብራርቶልኛል፡፡

**አግኝቻለሁ / አላገኘሁም፤** ስለ ጥናቱ ተገቢውን መግለጫ ማግኘቴን (ያክብቡ)

**ተረድቻለሁ / አልተረዳሁም፤** በተለያዩ ጊዜያትከልጄ ስለሚወሰዱ ናሙናና ስለጥናቱ ተጨባጭ ውጤት (ያክብቡ)

**ተስማምቻለሁ / አልተስማማሁም**፤ የተወሰነ ናሙና እስከ ጥናቱ መጨረሻ ጊዜ ድረስ እንዲቀመጥና ምርምር እንዲደረግበት (ያክብቡ)።

**ተስማምቻለሁ / አልተስማማሁም**፤ መጠይቁን ለማሟላት የቃል ጥያቄ ለማድረግ (ያክብቡ)

**ተስማምቻለሁ / አልተስማማሁም**፤ በጥናቱ ልጄ ለመሳተፉ (ያክብቡ)

መፃፍ ለማይችሉ የጣት እሻራ አዚህ ያሳርፉ

ወላጅ/ያሳዳጊ

ገለልተኛ ምስክር

የተሳታፊ ስም፦ ______________________________________

የተሳታፊ ቤተሰብ ስም፦ ______________________________________

የተሳታፊ ቤተሰብ ፊርማ፦ _______________ቀን፦________________

የገለልተኛ ምስክር ስም፦ _________________________________________________

የገለልተኛ ምስክር ፊርማ፦ ____________________ቀን________________

የተመራማሪው/ዋሰም፦_________________የተመራማሪው/ዋ ፊርማ፦_________ቀን፦__________

Amharic version of consent form III

# Appendix 5: Amharic version of information sheet II

**የጥናት መረጃ ቅጽ**

**ለቤተሰብ ወይም ለአሳዳጊ መረጃ ዕድሜያቸው ከ12-17**

**የጥናቱ ርዕስ፡** የፈልሲፓረም ወባ መድሃኒቶች ፈዋሽነት ማረጋገጫ ጥናት

ዋና ተመራማሪ፡ ዶ/ር እንዳለማው ጋዲሳ(ከአርማወር ሀንሰን የምርምር ተቋም)

የጥናቱ ድጋፍ፡ የኢትዮጲያ ጤና ጥበቃ ሚኒስተር

**መግቢያ**

እንደምን አደራችሁ/ዋላችሁ፡ስሜ------------------------------------እኔ በአርማወር ሀንሰን የምርምር ተቀም በወባ እና ትኩረት የሚሹ የሀሩር በሽታ የጥናት ቡድን አባል ነኝ፡፡ እኛ አሁን የፈለሲፓረም ወባ መድሃኒቶች ፈዋሽነት ማረጋገጫ ጥናት በኢትዮጲያ ማጥናት ይሆናል፡፡ይህን ጥናት በአርማወር ሀንሰን የምርምር ተቀም እና በፌደራል ጤና ጥበቃ ሚኒስተር ጋር በመተባበር ይሆናል፡፡ስለሆነም ልጅዎ በጥናቱ እንዲሳተፍ የእርሶን ይሁንታ እንጠይቃለን ምክንያቱም በልጁዎ ደሞ ውስጥ የወባ አምጪ ተዋሲያን ሰስለተገኘ ነው፤ጥናቱ በፍጹም ፈቃደኝነት ላይ የተመሰረተ ነው፡፡

**የጥናቱ ዓላማ**

ባለፉት አስር ዓመታት ተጨባጭ የሆነ የወባ ስርጭትና በወባ ምክንያት የሚከሰት ሞት መቀነስ ታይቷል። ይሁን እንጂ ይህ ስኬት ቀጣይ እንደሆነና የወባ በሽታ ከኢትዮጵያ ለማስወገድ አንዱ ፈታኝ ሁኔታ የወባ በሽታ አምጪ ተዋሲያን አሁን ያሉት ጸረ-ወባ መጋኒቶች የማዳን አቅም እየተዳከመ መምጣቱን ተከትሎ የማዳን አቅም ከፍ ለማድረግ እነዲያስችል አሁን ካሉት የመጀመሪያ ደረጃ ፀረ ወባ መድሐኒቶች ጋር ተጨማሪ የፀረ ወባ መድሐኒቶች በጥምረት በመጠቀም የማዳን አቅም እና የጎኑዮሽ ጉዳት ማጥናት ይሆናል፡፡ ስለሆነም ይህ ጥናት ዓላማ አርተሜሲኒን ሉፋትሪን ከ ፕሪማከን ጋር ላልተወሳሰበ ፕላስሞዲየም ፈልስፋረምን የማዳን አቅም መፈተሸ ይሆናል፡፡ በአሁኑ ጊዜ ላሉት የመጀመሪያ ደረጃ የፀረ ወባ መድሃኒቶችን ከተጨማሪ የጸረ-ወባ መዳኒቶች ጋር በማጣመር ውጤታማነቱን መገምገም ነው::

በመሆኑም ይህ ጥናት ሃገሪቱ ላቀደችው ወባን ፈጽሞ የማጥፋት አላማ ጋር ተያይዞ የፀረ ወባ መዳኒቶች የማዳን አቅም በየጊዜው መፈተሽ አስፈላጊነቱን ማጥናት እና ጥሩ እና ወሳኝ ግብአት እንዲሆን ማድረግ ይሆናል። ከዚህም የሚገኘው መረጃ ከሃገራችን ኢትዮጵያም አልፎ ተመሳሳይ ሁኔታ ላይ ላሉ ሌሎች ሃገራትም ጠቀሜታው የጎላ ይሆናል። ማንኛውም ጥናት ተያያዥ የሆነ የምቾት መጓደል ሊያመጣ ይችላል። ከዚህም ተያይዞ የሚቻለንን ያህል ሊኖሩ የሚችሉትን የምቾት መጓደሎች ለመቀነስ ጥረት እናደርጋለን። በማንኛውም ደረጃ አለም አቀፍ እና እንዲሁም በሃገራችን የተደነገጉ ህግጋትን ጠብቀን የህክምና ስነ-ምግባር በሚፈቅደው መሰረት እንሰራለን።

ስለሆነም ልጅዎ በዚህ ጥናት እንዲሳተፍ እንዲፈቅዱልን እንጠይቅዎታለን። ከልጅዎ የሚሰበሰበው የደም ናሙና ወደፊት ከወባ መስፋፋት ጋር ብቻ ለተያያዙ ጥናቶች ጥቅም ላይ ይውላል። በናሙናዎቹ ላይ ተጨማሪ ጥናት በሚያስፈልግበት ሁኔታ ከሚመለከተው አካል ፍቃድ እንጠይቃለን፣ እንደ ስምና አድራሻ ያሉ ግለሰባዊ መረጃዎች ከመረጃው ይነጠላሉ፤ የጥናት የስነምግባር ይሁንታም ይጠየቅባቸዋል።

**ጥናቱን ለመሳተፍ ቅድመ ሁኔታ**

ልጅዎ ይህን ጥናት ለመሳተፍ በመጀመሪያ በልጅዎ ደም ውስጥ የወባ አምጪ ተዋሲያሰን መገኘት አለበት፡፡

**ለጥናቱ የሚያስፈልጉ ተሳታፊዎች**

በአጠቃላይ 88 የፋልሲፓረም የወባ ተዋሲያን በደማቸው የተገኘባቸው ሰዎች በጥናቱ ይሳተፋሉ፡፡

**የጥናቱ ሂደት**

ውድ የጥናቱ ተሳታፊ፡ይህን የመረጃ ቅጽ ለመሙላት ወደ 20 ደቂቃ ይፈጃል። ላሳዩት ትግስት እና ልጅዎ በጥናቱ ለመሳተፉ በመፍቀድዎ አስቀድመን ልናመሰግንዎት እንወዳልን።

ልጅዎ በጥናቱ እንዲሳተፍ የእርስዎን ፍቃደኝነት እንጠይቃለን። ለዚህም እንዲረዳን የተለያዩ ምርመራዎችን ለማድረግ የሚረዳ ከደምስሩ 5 ሚሊ ሌትር የደም ናሙና በጥናቱ መጀመሪያ ላይ እንዲሰጡን ይጠየቃሉ፡፡ ከዚህ በኋላ ለተከታታይ ቀናቶች ከጣት ላይ(በ 0, 2, 3, 7, 10, 14, 21, 28, 42 የደም ናሙና ልጅዎ እንዲሰጥ እንጠይቃለን:: በጥናቱ ወቅት የምንወስደው የደም መጠን ከ 1ሚሊ እና ከ 5 ሚሊ መካከል ይገኛል፡፡ ይህ የደም መጠን በ 2 ወር ጊዜ ውስጥ በመከፋፈል የሚወሰድ ይሆናል፡፡ ለዚህም ይረዳ ዘንድ በቅድሚያ በመረዳት ላይ የተመረኮዘ ተሳትፎ እንዲያደርጉ በጥናቱ ለመሳተፍ ፍቃድዎን እነዲገልጹልን እንጠይቃለን፡፡

ልጁዎ የሰውነት ሙቀት መጨመር እና የወባ በሽታ ተመርምሮ ከተገኘበት ወዲያውኑ ህክምና ያገኛል፡፡ ከዚያም ለተከታታይ ቀናት (0, 1 2, 3, 7, 10, 14, 21, 28, 42) ክትትል ይደረጋል፡፡

**በፈቃደኝነት ላይ የተመሰረተ ተሳትፎ**

ልጅዎ በጥናቱ እንዳይሳተፍ ወይም እንዲያቋርጥ መወሰንዎ ልጅዎም ሆነ እርሶ ሊያገኙ የሚችሉት የጤና አገልግሎት ላይ ምንም ተፅእኖ አይኖረውም። ልጅዎ በጥናቱ እንዳይሳተፍ በማንኛውም ጊዜ እንዲያቋርጥ ማድረግ ይችላሉ። ልጅዎ በጥናቱ እንዳይሳተፍ ቢወስኑም እንኳን ተገቢውን አገልግሎት ያገኛሉ።

**የምቾት መጓደልና ተጋላጭለት**

ከልጅዎ የደም ናሙና በሚወሰድበት ጊዜ የተወሰነ የምቾት መጓደል ሊኖር ይችላል፤ ደም በተወሰደበትም ቦታ የተወሰነ የመቅላት ወይም መድማት ሊኖር ይችላል። ይህ ክስተት ግን ጎጂ እንዳልሆነ ይታሰባል። ናሙናዎችን ለመውሰድ ከጀርም የጸዱ መገልገያዎችን እንጠቀማለን፤ ከናሙና መውሰድ ጋር በተገናኘ ሊከሰት የሚችልን መጠነኛ ቁስለት ቢኖር እንኳን በተገቢው እናክማለን። የሚወሰደው የደም መጠን በጤና ላይ ተጽኖ ለማስከትል በጣም ትንሽ ነው፤ የተወሰደው ደምም በሰውነት በቶሎ ይተካል።

**ጠቀሜታ**

ልጅዎ በጥናቱ ጊዜ ከወባ ጋር የተያያዘ ነፃ የህክምና አገልግሎት ያገኛል/ታገኛለች። የተለየ አትኩሮት የሚፈልጉ ሁኔታዎች ከተከሰቱ በጤና ተቋም ህክምና እንዲያገኝ ይደረጋል። እርሶም ሆኑ ልጅዎ በጥናቱ በመሳተፋችሁ እና ላጠፉት ጊዜ ወይም ለትራንስፖርት እንደማካካሻ የምታገኙት 100 ብር ክፍያ ይኖራል፡፡

**በጥናቱ የመሳተፍ ነጻነት**

ጥናቱ በሙሉ ፈቃደኝነት ላይ የተመሰረተ ነው፡፡ ልጅዎ በጥናቱ እንዲሳተፍ ከወሰኑ መሳተፍ ይችላሉ፡ነገር ግን ልጅዎ በማንኛውም ጊዜ ከጥናቱ መውጣት ከፈለገ/ች መውጣት ይችላሉ፡፡ለዚህም በእርሶም ሆነ በልጅዎ ላይ የሚደርስ ቅጣት የለም፡፡

**ናሙናዎችን ጥናቱ ጊዜ እስከሚያልቅ ስለማስቀመጥ**

ከእርሶና ከልጅዎ የሚገኘውን ናሙና የጥናቱ ጊዜ እስከሚያልቅ ማስቀመጥ እንድንችል ፈቃድዎትን እንጠይቃለን። ናሙናዎች የሚቀመጡት የጥናቱ ጊዜ እስኪጠናቀቅ ጊዜ በቻ ይሆናል፡፡ ናሙናዎችን የምንጠቀማቸው የወባ ጸረ-መዳኒቶች ላይ ያላቸውን አቅም ከመፈተሸ ጋር በተዛመደ ጥናት፤ የሚረዱ መረጃዎችን ለማግኘት ብቻ ይሆናል። ተጨማሪ ጥናት አስፈላጊ ከሆነ የስነምግባር ኮሚቴ ይሁንታን የሚጠየቅበት ይሆናል።

**ሚስጥርን ስለመጠበቅ**

ከልጅዎ መሳትፍ የሚገኙት መረጃዎች ለዚህ ጥናት ጠቀሜታ ብቻ ይውላሉ። የልጅዎ ስም ለናሙና መለያነት ወይም በማንኛውም የዚህ ጥናት ውጤት ሪፖርት ላይ አንጠቀምም። በጥናቱ መጀመሪያ ለተሳታፊዎች የመለያ የሚስጥር ቁጥር ይሰጣል፤ ይህም ለናሙናዎችና በጥናቱ በጥቅም ላይ ለሚውሉ ቅጾች መለያነት ይጠቅማል። ማንኛውም ከዚህ ጥናት በተዛመደ የሚገኝ መረጃ በሚስጥር ይያዛል፤ መረጃዎችም በቁልፍ ተቆልፎባቸው ይቀመጣሉ። የልጅዎ ስም ከጥናቱ የሚስጥር ቁጥር ጋር የሚያገናኘውን መረጃ የጥናቱ ዋና ተመራማሪዎች ብቻ ናቸው ማየት የሚችሉት።

**ጥያቄዎችን የመጠየቅና ከጥናቱ አቋርጦ የመውጣት ነጻነት**

ልጅዎ ጥናቱን በተመለከተ ጥያቄ ካላቸው፤ የጥናቱን ዋና ተመራማሪ ዶ/ር እንዳላማው ጋዲሳን ለመጠየቅ አያመንቱ። አርማወር ሀንሰን የምርምር ተቋም, ፖሰት ሳጥን ቁጥር 1005, አዲስ አበባ, ኢትዮጵያ ስልክ ቁጥር፦0911868827)። ስለጥናቱ እና ከጥናቱ ጋር ያልተገናኘ ገለልተኛ ወገንን ማማከር ካስፈለጋችሁ እንደጥናቱ ተሳታፊነታችሁ እርሶም ሆኑ ልጅዎት የአርማዉር ሃንሰን ምርምር ተቋም/ የአለርት የስነ-ምግባር ኮሚቴ ጸሃፊን በተመለከተው አድራሻ ማግኘት ይችላሉ (0118-962183)።

Appendix 6: Amharic version of consent form I

**ለወላጅ/አሳዳጊ የስምምነት መግለጫ ቅጽ ዕድሜያቸው 12-17 ዓመት**

**የጥናቱ ርእስ፡-** የፈለሲፓረም ወባ መድሃኒቶች ፈዋሽነት ማረጋገጫ ጥናት

እኔ ወላጅ/አሳዳጊ, ልጄ በፈቃደኛነት በጥናት ተሳታፊ እንዲሆን የፈቀድኩት የተሳታፊነት ማሳወቂያ ቅፁን ከመፈረሜ አስቀድሞ ሥለጥናቱ አካሄድ እና ከዚህ ቀጥሎ የተዘረዘሩትን የጥናቱ ተሳታፊ መብቶች ግልፅ በሆነ መልኩ ከመረጃ ቅፁ አንብቤ ተረድቻለሁ /ተነቦልኝ በግልፅ ተብራርቶልኛል፡፡

**አግኝቻለሁ / አላገኘሁም፤** ስለ ጥናቱ ተገቢውን መግለጫ ማግኘቴን (ያክብቡ)

**ተረድቻለሁ / አልተረዳሁም፤** በተለያዩ ጊዜያት የልጄን በመብጣት እና ከክንዱ ላይ ስለሚወሰድ ናሙናና ስለጥናቱ ተጨባጭ ውጤት (ያክብቡ)

**ተስማምቻለሁ / አልተስማማሁም**፤ የተወሰነ ናሙና እስከ ጥናቱ መጨረሻ ጊዜ ድረስ እንዲቀመጥና ምርምር እንዲደረግበት (ያክብቡ)።

**ተስማምቻለሁ / አልተስማማሁም**፤ መጠይቁን ለማሟላት የቃል ጥያቄ ለማድረግ (ያክብቡ)

**ተስማምቻለሁ / አልተስማማሁም**፤ በጥናቱ ልጄ ለመሳተፉ (ያክብቡ)

መፃፍ ለማይችሉ የጣት እሻራ አዚህ ያሳርፉ

ወላጅ/ያሳዳጊ

ገለልተኛ ምስክር

የተሳታፊ ስም፦ ______________________________________

የተሳታፊ ፊርማ-------------------------------ቀን------------------

የተሳታፊ ቤተሰብ ስም፦ ______________________________________

የተሳታፊ ቤተሰብ ፊርማ፦ _______________ቀን፦________________

የገለልተኛ ምስክር ስም፦ _________________________________________________

የገለልተኛ ምስክር ፊርማ፦ ____________________ቀን________________

የተመራማሪው/ዋሰም፦_________________የተመራማሪው/ዋ ፊርማ፦_________ቀን፦__________

**የጥናት መረጃ ቅጽ**

**ጥናቱ ተሳታፊ የመረጃ ፎርም ዕድሜያቸው ከ ≥18 ዓመት በላይ ለሆኑ ተሳታፊዎች**

**ለተሳታፊዎች መረጃ**

**የጥናቱ ርዕስ፡** የቫይቫክስ ወባ መድሃኒቶች ፈዋሽነት ማረጋገጫ ጥናት

ዋና ተመራማሪ፡ ዶ/ር እንዳለማው ጋዲሳ(ከአርማወር ሀንሰን የምርምር ተቋም)

የጥናቱ በጀት ደጋፊ፡ የኢትዮጲያ ጤና ጥበቃ ሚኒስተር

**መግቢያ**

እንደምን አደራችሁ/ዋላችሁ፡ስሜ------------------------------------እኔ በአርማወር ሀንሰን የምርምር ተቀም በወባ እና ትኩረት የሚሹ የሀሩር በሽታዎች የጥናት ቡድን አባል ነኝ፡፡ እኛ አሁን የጥምረት የጸረ-ወባ መዳኒት የፈዋሽኝነት አቅም በኢትዮጲያ ማጥናት ይሆናል፡፡ይህን ጥናት በፌደራል ጤና ጥበቃ ሚኒስተር ይደገፋል፡፡ ስለሆነም እርሶ ወይም ልጅዎ በጥናቱ እንዲሳተፉ እንጠይቃለን ምክንያቱም በደሞ ውስጥ የወባ አምጪ ተዋሲያን ሰስለተገኘ ነው፤ጥናቱ በፍጹም ፈቃደኝነት ላይ የተመሰረተ ነው፡፡

**የጥናቱ ዓላማ**

ባለፉት አስር ዓመታት ተጨባጭ የሆነ የወባ ስርጭትና በወባ ምክንያት የሚከሰት ሞት መቀነስ ታይቷል። ይሁን እንጂ ይህ ስኬት ቀጣይ እንደሆነና የወባ በሽታ ከኢትዮጵያ ለማስወገድ አንዱ ፈታኝ ሁኔታ የወባ በሽታ አምጪ ተዋሲያን አሁን ያሉት ጸረ-ወባ መጋኒቶች የማዳን አቅም እየተዳከመ መምጣቱን ተከትሎ የማዳን አቅም ከፍ ለማድረግ እነዲያስችል አሁን ካሉት የመጀመሪያ ደረጃ ፀረ ወባ መድሐኒቶች ጋር ተጨማሪ የፀረ ወባ መድሐኒቶች በጥምረት በመጠቀም የማዳን አቅም እና የጎኑዮሽ ጉዳት ማጥናት ይሆና፡፡ ስለሆነም ይህ ጥናት ዓላማ ክሎረኪን ከ 14 ቀን ፕሪማከን ጋር ላልተወሳሰበ ፕላስሞዲየም ፈልስፋረምን የማዳን አቅም መፈተሸ ይሆናል፡፡

በመሆኑም ይህ ጥናት ሃገሪቱ ላቀደችው ወባን ፈጽሞ የማጥፋት አላማ ጋር ተያይዞ የፀረ ወባ መዳኒቶች የማዳን አቅም በየጊዜው መፈተሽ አስፈላጊነቱን ማጥናት እና ጥሩ እና ወሳኝ ግብአት እንዲሆን ማድረግ ይሆናል። ከዚህም የሚገኘው መረጃ ከሃገራችን ኢትዮጵያም አልፎ ተመሳሳይ ሁኔታ ላይ ላሉ ሌሎች ሃገራትም ጠቀሜታው የጎላ ይሆናል። ማንኛውም ጥናት ተያያዥ የሆነ የምቾት መጓደል ሊያመጣ ይችላል። ከዚህም ተያይዞ የሚቻለንን ያህል ሊኖሩ የሚችሉትን የምቾት መጓደሎች ለመቀነስ ጥረት አድርገናል። በማንኛውም ደረጃ አለም አቀፍ እና እንዲሁም በሃገራችን የተደነገጉ ህግጋትን ጠብቀን የህክምና ስነ-ምግባር በሚፈቅደው መሰረት እንሰራለን።

ስለሆነም እርሶ ወይም ልጅዎ በዚህ ጥናት ለመሳተፍ ፈቃደዎን እነዲገልጹልን እንጠይቅዎታለን። ከእርሶ/ከልጅዎ የሚሰበሰበው የደም ናሙና ወደፊት ከወባ መስፋፋት ጋር ብቻ ለተያያዙ ጥናቶች ጥቅም ላይ ይውላል። በናሙናዎቹ ላይ ተጨማሪ ጥናት በሚያስፈልግበት ሁኔታ ከሚመለከተው አካል ፍቃድ እንጠይቃለን፣ እንደ ስምና አድራሻ ያሉ ግለሰባዊ መረጃዎች ከመረጃው ይነጠላሉ፤ የጥናት የስነምግባር ይሁንታም ይጠየቅባቸዋል።

**ጥናቱን ለመሳተፍ ቅድመ ሁኔታ**

እርሶ ወይም ልጅዎ ይህን ጥናት ለመሳተፍ በመጀመሪያ በእርሶ ወይም በልጅዎ ደም ውስጥ የወባ አምጪ ተዋሲያሰን መገኘት አለበት፡፡

**ለጥናቱ የሚያስፈልጉ ተሳታፊዎች**

በአጠቃላይ 88 የቫይቫክስ የወባ ተዋሲያን በደማቸው የተገኘባቸው ሰዎች በጥናቱ ይሳተፋሉ፡፡

**የጥናቱ ሂደት**

ውድ የጥናቱ ተሳታፊ፡ይህን የመረጃ ቅጽ ለመሙላት ወደ 20 ደቂቃ ይፈጃል። ላሳዩት ትግስት እናእርሶ/ ልጅዎ በጥናቱ ለመሳተፉ በመፍቀድዎ አስቀድመን ልናመሰግንዎት እንወዳልን።

እርሶ/ልጅዎ በጥናቱ ለመሳተፍ የእርስዎን ፍቃደኝነት እንጠይቃለን። ለዚህም እንዲረዳን የተለያዩ ምርመራዎችን ለማድረግ የሚረዳ 5 ሚሊ ሌትር የደም ናሙና በጥናቱ መጀመሪያ ላይ እንዲሰጡን ይጠየቃሉ፡፡ ከዚህ በኋላ ለተከታታይ ቀናቶች ከጣት ላይ(በ 0, 2, 3, 7, 10, 14, 21, 28, 42 የደም ናሙና እርሶ/ልጅዎ እንዲሰጡ እንጠይቃለን:: በጥናቱ ወቅት የምንወስደው የደም መጠን ከ 1ሚሊ እና 5 ሚሊ መካከል ይገኛል፡፡ ይህ የደም መጠን በ 2 ወር ጊዜ ውስጥ በመከፋፈል የሚወሰድ ይሆናል፡፡ ለዚህም ይረዳ ዘንድ በቅድሚያ በመረዳት ላይ የተመረኮዘ ተሳትፎ እንዲያደርጉ በጥናቱ ለመሳተፍ ፍቃድዎን እንዲገልጹልን እንጠይቃለን፡፡

ሁሉም ሰው የሰውነት ሙቀት መጨመር እና የወባ በሽታ ተመርምሮ ከተገኘበት ወዲያውኑ ህክምና ያገኛል፡፡ ከዚያም ለተከታታይ ቀናት (0, 1 2, 3, 7, 10, 14, 21, 28, 42) ክትትል ይደረጋል፡፡

**በፈቃደኝነት ላይ የተመሰረተ ተሳትፎ**

እርሶ/ልጅዎ በጥናቱ እንዳይሳተፍ ወይም እንዲያቋርጥ መወሰንዎ ልጅዎም ሆነ እርሶ ሊያገኙ የሚችሉት የጤና አገልግሎት ላይ ምንም ተፅእኖ አይኖረውም። እርሶ/ልጅዎ ጥናቱን ማረጥ ከፈለጋችሁ በማንኛውም ጊዜ ማቋረጥ ይችላሉ። እርሶ/ልጅዎ በጥናቱ እንዳይሳተፍ ቢወስኑም እንኳን ተገቢውን አገልግሎት ያገኛሉ።

**የምቾት መጓደልና ተጋላጭለት**

ከእርሶ/ልጅዎ የደም ናሙና በሚወሰድበት ጊዜ የተወሰነ የምቾት መጓደል ሊኖር ይችላል፤ ደም በተወሰደበትም ቦታ የተወሰነ የመቅላት ወይም መድማት ሊኖር ይችላል። ይህ ክስተት ግን ጎጂ እንዳልሆነ ይታሰባል። ናሙናዎችን ለመውሰድ ከጀርም የጸዱ መገልገያዎችን እንጠቀማለን፤ ከናሙና መውሰድ ጋር በተገናኘ ሊከሰት የሚችልን መጠነኛ ቁስለት ቢኖር እንኳን በተገቢው እናክማለን። የሚወሰደው የደም መጠን በጤና ላይ ተጽኖ ለማስከትል በጣም ትንሽ ነው፤ የተወሰደው ደምም በሰውነት በቶሎ ይተካል።

**ጠቀሜታ**

እርሶ/ልጅዎ በጥናቱ ጊዜ ከወባ ጋር የተያያዘ ነፃ የህክምና አገልግሎት ታገኛለችሁ። የተለየ አትኩሮት የሚፈልጉ ሁኔታዎች ከተከሰቱ በጤና ተቋም ህክምና እንዲያገኙ ይደረጋል። እርሶም ሆኑ ልጅዎ በጥናቱ በመሳተፋችሁ እና ላጠፉት ጊዜ ወይም ለትራንስፖርት እንደማካካሻ የምታገኙት 100 ብር ክፍያ ይኖራል፡፡

**በጥናቱ የመሳተፍ ነጻነት**

ጥናቱ በሙሉ ፈቃደኝነት ላይ የተመሰረተ ነው፡፡እርሶ ወይም ልጅዎ በጥናቱ ለመሳተፍ ከወሰኑ መሳተፍ ይችላሉ፡ነገር ግን እርሶ ወይም ልጅዎ በማንኛውም ጊዜ ከጥናቱ መውጣት ከፈለጉ መውጣት ይችላሉ፡፡ለዚህም በእርሶም ሆነ በልጅዎ ላይ የሚደርስ ቅጣት የለም፡፡

**ናሙናዎችን ጥናቱ ጊዜ እስከሚያልቅ ስለማስቀመጥ**

ከእርሶና ከልጅዎ የሚገኘውን ናሙና የጥናቱ ጊዜ እስከሚያልቅ ማስቀመጥ እንድንችል ፈቃድዎትን እንጠይቃለን። ናሙናዎች የሚቀመጡት የጥናቱ ጊዜ እስኪጠናቀቅ ጊዜ በቻ ይሆናል፡፡ ናሙናዎችን የምንጠቀማቸው የወባ ጸረ-መዳኒቶች ላይ ያላቸውን አቅም ከመፈተሸ ጋር በተዛመደ ጥናት፤ የሚረዱ መረጃዎችን ለማግኘት ብቻ ይሆናል። ተጨማሪ ጥናት አስፈላጊ ከሆነ የስነምግባር ኮሚቴ ይሁንታን የሚጠየቅበት ይሆናል።

**ሚስጥርን ስለመጠበቅ**

ከእርሶ ወይም ከልጅዎ መሳትፍ የሚገኙት መረጃዎች ለዚህ ጥናት ጠቀሜታ ብቻ ይውላሉ። የርሶም ሆነ የልጅዎ ስም ለናሙና መለያነት ወይም በማንኛውም የዚህ ጥናት ውጤት ሪፖርት ላይ አንጠቀምም። በጥናቱ መጀመሪያ ለተሳታፊዎች የመለያ የሚስጥር ቁጥር ይሰጣል፤ ይህም ለናሙናዎችና በጥናቱ በጥቅም ላይ ለሚውሉ ቅጾች መለያነት ይጠቅማል። ማንኛውም ከዚህ ጥናት በተዛመደ የሚገኝ መረጃ በሚስጥር ይያዛል፤ መረጃዎችም በቁልፍ ተቆልፎባቸው ይቀመጣሉ። የእርስዎንም ሆነ የልጅዎን ስም ከጥናቱ የሚስጥር ቁጥር ጋር የሚያገናኘውን መረጃ የጥናቱ ዋና ተመራማሪዎች ብቻ ናቸው ማየት የሚችሉት።

**ጥያቄዎችን የመጠየቅና ከጥናቱ አቋርጦ የመውጣት ነጻነት**

ከጥናቱ ጋር በተያያዘ ጥያቄ ካላችሁ፤ የጥናቱን ዋና ተመራማሪ ዶ/ር እንዳላማው ጋዲሳን ለመጠየቅ አያመንቱ። አርማወር ሀንሰን የምርምር ተቋም, ፖሰት ሳጥን ቁጥር 1005, አዲስ አበባ, ኢትዮጵያ ስልክ ቁጥር፦0911868827)። ስለጥናቱ እና ከጥናቱ ጋር ያልተገናኘ ገለልተኛ ወገንን ማማከር ካስፈለጋችሁ እንደጥናቱ ተሳታፊነታችሁ የአርማዉር ሃንሰን ምርምር ተቋም/ የአለርት የስነ-ምግባር ኮሚቴ ጸሃፊን በተመለከተው አድራሻ ማግኘት ይችላሉ (0118-962183)።

**የጥናቱ ተሳታፊ የስምምነት መግለጫ ቅጽ ዕድሜያቸው ከ 18 ዓመት በላይ ለሆኑ ተሳታፊዎች**

እኔ ___________________________________ባለኝ ሙሉ ሃላፊነት ጥናት ውስጥ እንድሳተፍ ተስማምቻለሁ። በጥነቱ ተሳታፊ የሆንኩት በገዛ ፍቃዴ ነዉ፡፡ ያለምንም ቅጣት በማንኛውም የጥናቱ ወቅት ፈቃዴን ማንሳትና ከጥናቱ መገለል እንደምችል ተረድቻለሁ። ሰለሆነም በጥናቱ ለመሳተፍ ፍቃደኝነቴን በፊርማየ አረጋግጣለሁ፡፡ የስምምነት መግለጫ ቅጹ ግልባጭ ይሰጠወታል፡፡

ፊርማ|አሻራ፦ ____________ ቀን፦ ________________

የተሳታፊው ሰም፦ ___________________________

መፈረም ለማይችሉ፤

ተሳታፊዉ የስምምነት መግለጫ ቅጹ ተነቦለት በጥናቱ ለመሳተፍ ፍቃደኝነቱን መግለጹን እመሰክራለሁ፡፡

የምስክር ስም፦_______________________ፊርማ|አሻራ፦____________ ቀን፦ ___________

ናሙናን ለማስቀመጥ ፈቃደኝነት (ፊርማ|አሻራ)፡ የተወሰደው የደም ናሙና ለወደፊት በወባ ላይ ተመሳሳይ ስራ እንዲሰራበት ፈቃደኛ ነኝ

ፊርማ|አሻራ፦ ____________ ቀን፦ ________________

የተሳታፊው ሰም፦ ___________________________

መፈረም ለማይችሉ፤

ተሳታፊዉ የስምምነት መግለጫ ቅጹ ተነቦለት በጥናቱ ለመሳተፍ ፍቃደኝነቱን መግለጹን እመሰክራለሁ፡፡

የምስክር ስም፦_______________________ፊርማ|አሻራ፦____________ ቀን፦ ___________

**ለወላጅ/አሳዳጊ የስምምነት መግለጫ ቅጽ ዕድሜያቸው <18 ዓመት**

**የጥናቱ ርእስ፡-** የቫይቫክስ ወባ መድሃኒቶች ፈዋሽነት ማረጋገጫ ጥናት

እኔ ወላጅ/አሳዳጊ, በጥናቱ ልጄ እንዲሳተፍ የተሳታፊነት ማሳወቂያ ቅፁን ከመፈረሜ አስቀድሞ ሥለጥናቱ አካሄድ እና ከዚህ ቀጥሎ የተዘረዘሩትን የጥናቱ ተሳታፊ መብቶች ግልፅ በሆነ መልኩ ከመረጃ ቅፁ አንብቤ ተረድቻለሁ /ተነቦልኝ በግልፅ ተብራርቶልኛል፡፡

**አግኝቻለሁ / አላገኘሁም፤** ስለ ጥናቱ ተገቢውን መግለጫ ማግኘቴን (ያክብቡ)

**ተረድቻለሁ / አልተረዳሁም፤** በተለያዩ ጊዜያትከልጄ ስለሚወሰዱ ናሙናና ስለጥናቱ ተጨባጭ ውጤት (ያክብቡ)

**ተስማምቻለሁ / አልተስማማሁም**፤ የተወሰነ ናሙና እስከ ጥናቱ መጨረሻ ጊዜ ድረስ እንዲቀመጥና ምርምር እንዲደረግበት (ያክብቡ)።

**ተስማምቻለሁ / አልተስማማሁም**፤ መጠይቁን ለማሟላት የቃል ጥያቄ ለማድረግ (ያክብቡ)

**ተስማምቻለሁ / አልተስማማሁም**፤ በጥናቱ ልጄ ለመሳተፉ (ያክብቡ)

መፃፍ ለማይችሉ የጣት እሻራ አዚህ ያሳርፉ

ወላጅ/ያሳዳጊ

ገለልተኛ ምስክር

የተሳታፊ ስም፦ ______________________________________

የተሳታፊ ቤተሰብ ስም፦ ______________________________________

የተሳታፊ ቤተሰብ ፊርማ፦ _______________ቀን፦________________

የገለልተኛ ምስክር ስም፦ _________________________________________________

የገለልተኛ ምስክር ፊርማ፦ ____________________ቀን________________

የተመራማሪው/ዋሰም፦_________________የተመራማሪው/ዋ ፊርማ፦_________ቀን፦__________

# Amharic version of consent form III

# Appendix 5: Amharic version of information sheet II

**የጥናት መረጃ ቅጽ**

**ለቤተሰብ ወይም ለአሳዳጊ መረጃ ዕድሜያቸው ከ12-17**

**የጥናቱ ርዕስ፡** የቫይቫክስ ወባ መድሃኒቶች ፈዋሽነት ማረጋገጫ ጥናት

ዋና ተመራማሪ፡ ዶ/ር እንዳለማው ጋዲሳ(ከአርማወር ሀንሰን የምርምር ተቋም)

የጥናቱ ድጋፍ፡ የኢትዮጲያ ጤና ጥበቃ ሚኒስተር

**መግቢያ**

እንደምን አደራችሁ/ዋላችሁ፡ስሜ------------------------------------እኔ በአርማወር ሀንሰን የምርምር ተቀም በወባ እና ትኩረት የሚሹ የሀሩር በሽታ የጥናት ቡድን አባል ነኝ፡፡ እኛ አሁን የየቫይቫክስ ወባ መድሃኒቶች ፈዋሽነት ማረጋገጫ ጥናት ወባ መድሃኒቶች ፈዋሽነት ማረጋገጫ ጥናት በኢትዮጲያ ማጥናት ይሆናል፡፡ይህን ጥናት በአርማወር ሀንሰን የምርምር ተቀም እና በፌደራል ጤና ጥበቃ ሚኒስተር ጋር በመተባበር ይሆናል፡፡ስለሆነም ልጅዎ በጥናቱ እንዲሳተፍ የእርሶን ይሁንታ እንጠይቃለን ምክንያቱም በልጁዎ ደሞ ውስጥ የወባ አምጪ ተዋሲያን ሰስለተገኘ ነው፤ጥናቱ በፍጹም ፈቃደኝነት ላይ የተመሰረተ ነው፡፡

**የጥናቱ ዓላማ**

ባለፉት አስር ዓመታት ተጨባጭ የሆነ የወባ ስርጭትና በወባ ምክንያት የሚከሰት ሞት መቀነስ ታይቷል። ይሁን እንጂ ይህ ስኬት ቀጣይ እንደሆነና የወባ በሽታ ከኢትዮጵያ ለማስወገድ አንዱ ፈታኝ ሁኔታ የወባ በሽታ አምጪ ተዋሲያን አሁን ያሉት ጸረ-ወባ መጋኒቶች የማዳን አቅም እየተዳከመ መምጣቱን ተከትሎ የማዳን አቅም ከፍ ለማድረግ እነዲያስችል አሁን ካሉት የመጀመሪያ ደረጃ ፀረ ወባ መድሐኒቶች ጋር ተጨማሪ የፀረ ወባ መድሐኒቶች በጥምረት በመጠቀም የማዳን አቅም እና የጎኑዮሽ ጉዳት ማጥናት ይሆናል፡፡ ስለሆነም ይህ ጥናት ዓላማ ክለሎረኪን ከ14 ቀን ፕሪማከን ጋር ላልተወሳሰበ ፕላስሞዲየም ቫይቫክስ የማዳን አቅም መፈተሸ ይሆናል፡፡ በአሁኑ ጊዜ ላሉት የመጀመሪያ ደረጃ የፀረ ወባ መድሃኒቶችን ከተጨማሪ የጸረ-ወባ መዳኒቶች ጋር በማጣመር ውጤታማነቱን መገምገም ነው::

በመሆኑም ይህ ጥናት ሃገሪቱ ላቀደችው ወባን ፈጽሞ የማጥፋት አላማ ጋር ተያይዞ የፀረ ወባ መዳኒቶች የማዳን አቅም በየጊዜው መፈተሽ አስፈላጊነቱን ማጥናት እና ጥሩ እና ወሳኝ ግብአት እንዲሆን ማድረግ ይሆናል። ከዚህም የሚገኘው መረጃ ከሃገራችን ኢትዮጵያም አልፎ ተመሳሳይ ሁኔታ ላይ ላሉ ሌሎች ሃገራትም ጠቀሜታው የጎላ ይሆናል። ማንኛውም ጥናት ተያያዥ የሆነ የምቾት መጓደል ሊያመጣ ይችላል። ከዚህም ተያይዞ የሚቻለንን ያህል ሊኖሩ የሚችሉትን የምቾት መጓደሎች ለመቀነስ ጥረት እናደርጋለን። በማንኛውም ደረጃ አለም አቀፍ እና እንዲሁም በሃገራችን የተደነገጉ ህግጋትን ጠብቀን የህክምና ስነ-ምግባር በሚፈቅደው መሰረት እንሰራለን።

ስለሆነም ልጅዎ በዚህ ጥናት እንዲሳተፍ እንዲፈቅዱልን እንጠይቅዎታለን። ከልጅዎ የሚሰበሰበው የደም ናሙና ወደፊት ከወባ መስፋፋት ጋር ብቻ ለተያያዙ ጥናቶች ጥቅም ላይ ይውላል። በናሙናዎቹ ላይ ተጨማሪ ጥናት በሚያስፈልግበት ሁኔታ ከሚመለከተው አካል ፍቃድ እንጠይቃለን፣ እንደ ስምና አድራሻ ያሉ ግለሰባዊ መረጃዎች ከመረጃው ይነጠላሉ፤ የጥናት የስነምግባር ይሁንታም ይጠየቅባቸዋል።

**ጥናቱን ለመሳተፍ ቅድመ ሁኔታ**

ልጅዎ ይህን ጥናት ለመሳተፍ በመጀመሪያ በልጅዎ ደም ውስጥ የወባ አምጪ ተዋሲያሰን መገኘት አለበት፡፡

**ለጥናቱ የሚያስፈልጉ ተሳታፊዎች**

በአጠቃላይ 88 የቫይቫክስ የወባ ተዋሲያን በደማቸው የተገኘባቸው ሰዎች በጥናቱ ይሳተፋሉ፡፡

**የጥናቱ ሂደት**

ውድ የጥናቱ ተሳታፊ፡ይህን የመረጃ ቅጽ ለመሙላት ወደ 20 ደቂቃ ይፈጃል። ላሳዩት ትግስት እና ልጅዎ በጥናቱ ለመሳተፉ በመፍቀድዎ አስቀድመን ልናመሰግንዎት እንወዳልን።

ልጅዎ በጥናቱ እንዲሳተፍ የእርስዎን ፍቃደኝነት እንጠይቃለን። ለዚህም እንዲረዳን የተለያዩ ምርመራዎችን ለማድረግ የሚረዳ ከደምስሩ 5 ሚሊ ሌትር የደም ናሙና በጥናቱ መጀመሪያ ላይ እንዲሰጡን ይጠየቃሉ፡፡ ከዚህ በኋላ ለተከታታይ ቀናቶች ከጣት ላይ(በ 0, 2, 3, 7, 10, 14, 21, 28, 42 የደም ናሙና ልጅዎ እንዲሰጥ እንጠይቃለን:: በጥናቱ ወቅት የምንወስደው የደም መጠን ከ 1ሚሊ እና ከ 5 ሚሊ መካከል ይገኛል፡፡ ይህ የደም መጠን በ 2 ወር ጊዜ ውስጥ በመከፋፈል የሚወሰድ ይሆናል፡፡ ለዚህም ይረዳ ዘንድ በቅድሚያ በመረዳት ላይ የተመረኮዘ ተሳትፎ እንዲያደርጉ በጥናቱ ለመሳተፍ ፍቃድዎን እነዲገልጹልን እንጠይቃለን፡፡

ልጁዎ የሰውነት ሙቀት መጨመር እና የወባ በሽታ ተመርምሮ ከተገኘበት ወዲያውኑ ህክምና ያገኛል፡፡ ከዚያም ለተከታታይ ቀናት (0, 1 2, 3, 7, 10, 14, 21, 28, 42) ክትትል ይደረጋል፡፡

**በፈቃደኝነት ላይ የተመሰረተ ተሳትፎ**

ልጅዎ በጥናቱ እንዳይሳተፍ ወይም እንዲያቋርጥ መወሰንዎ ልጅዎም ሆነ እርሶ ሊያገኙ የሚችሉት የጤና አገልግሎት ላይ ምንም ተፅእኖ አይኖረውም። ልጅዎ በጥናቱ እንዳይሳተፍ በማንኛውም ጊዜ እንዲያቋርጥ ማድረግ ይችላሉ። ልጅዎ በጥናቱ እንዳይሳተፍ ቢወስኑም እንኳን ተገቢውን አገልግሎት ያገኛሉ።

**የምቾት መጓደልና ተጋላጭለት**

ከልጅዎ የደም ናሙና በሚወሰድበት ጊዜ የተወሰነ የምቾት መጓደል ሊኖር ይችላል፤ ደም በተወሰደበትም ቦታ የተወሰነ የመቅላት ወይም መድማት ሊኖር ይችላል። ይህ ክስተት ግን ጎጂ እንዳልሆነ ይታሰባል። ናሙናዎችን ለመውሰድ ከጀርም የጸዱ መገልገያዎችን እንጠቀማለን፤ ከናሙና መውሰድ ጋር በተገናኘ ሊከሰት የሚችልን መጠነኛ ቁስለት ቢኖር እንኳን በተገቢው እናክማለን። የሚወሰደው የደም መጠን በጤና ላይ ተጽኖ ለማስከትል በጣም ትንሽ ነው፤ የተወሰደው ደምም በሰውነት በቶሎ ይተካል።

**ጠቀሜታ**

ልጅዎ በጥናቱ ጊዜ ከወባ ጋር የተያያዘ ነፃ የህክምና አገልግሎት ያገኛል/ታገኛለች። የተለየ አትኩሮት የሚፈልጉ ሁኔታዎች ከተከሰቱ በጤና ተቋም ህክምና እንዲያገኝ ይደረጋል። እርሶም ሆኑ ልጅዎ በጥናቱ በመሳተፋችሁ እና ላጠፉት ጊዜ ወይም ለትራንስፖርት እንደማካካሻ የምታገኙት 100 ብር ክፍያ ይኖራል፡፡

**በጥናቱ የመሳተፍ ነጻነት**

ጥናቱ በሙሉ ፈቃደኝነት ላይ የተመሰረተ ነው፡፡ ልጅዎ በጥናቱ እንዲሳተፍ ከወሰኑ መሳተፍ ይችላሉ፡ነገር ግን ልጅዎ በማንኛውም ጊዜ ከጥናቱ መውጣት ከፈለገ/ች መውጣት ይችላሉ፡፡ለዚህም በእርሶም ሆነ በልጅዎ ላይ የሚደርስ ቅጣት የለም፡፡

**ናሙናዎችን ጥናቱ ጊዜ እስከሚያልቅ ስለማስቀመጥ**

ከእርሶና ከልጅዎ የሚገኘውን ናሙና የጥናቱ ጊዜ እስከሚያልቅ ማስቀመጥ እንድንችል ፈቃድዎትን እንጠይቃለን። ናሙናዎች የሚቀመጡት የጥናቱ ጊዜ እስኪጠናቀቅ ጊዜ በቻ ይሆናል፡፡ ናሙናዎችን የምንጠቀማቸው የወባ ጸረ-መዳኒቶች ላይ ያላቸውን አቅም ከመፈተሸ ጋር በተዛመደ ጥናት፤ የሚረዱ መረጃዎችን ለማግኘት ብቻ ይሆናል። ተጨማሪ ጥናት አስፈላጊ ከሆነ የስነምግባር ኮሚቴ ይሁንታን የሚጠየቅበት ይሆናል።

**ሚስጥርን ስለመጠበቅ**

ከልጅዎ መሳትፍ የሚገኙት መረጃዎች ለዚህ ጥናት ጠቀሜታ ብቻ ይውላሉ። የልጅዎ ስም ለናሙና መለያነት ወይም በማንኛውም የዚህ ጥናት ውጤት ሪፖርት ላይ አንጠቀምም። በጥናቱ መጀመሪያ ለተሳታፊዎች የመለያ የሚስጥር ቁጥር ይሰጣል፤ ይህም ለናሙናዎችና በጥናቱ በጥቅም ላይ ለሚውሉ ቅጾች መለያነት ይጠቅማል። ማንኛውም ከዚህ ጥናት በተዛመደ የሚገኝ መረጃ በሚስጥር ይያዛል፤ መረጃዎችም በቁልፍ ተቆልፎባቸው ይቀመጣሉ። የልጅዎ ስም ከጥናቱ የሚስጥር ቁጥር ጋር የሚያገናኘውን መረጃ የጥናቱ ዋና ተመራማሪዎች ብቻ ናቸው ማየት የሚችሉት።

**ጥያቄዎችን የመጠየቅና ከጥናቱ አቋርጦ የመውጣት ነጻነት**

ልጅዎ ጥናቱን በተመለከተ ጥያቄ ካላቸው፤ የጥናቱን ዋና ተመራማሪ ዶ/ር እንዳላማው ጋዲሳን ለመጠየቅ አያመንቱ። አርማወር ሀንሰን የምርምር ተቋም, ፖሰት ሳጥን ቁጥር 1005, አዲስ አበባ, ኢትዮጵያ ስልክ ቁጥር፦0911868827)። ስለጥናቱ እና ከጥናቱ ጋር ያልተገናኘ ገለልተኛ ወገንን ማማከር ካስፈለጋችሁ እንደጥናቱ ተሳታፊነታችሁ እርሶም ሆኑ ልጅዎት የአርማዉር ሃንሰን ምርምር ተቋም/ የአለርት የስነ-ምግባር ኮሚቴ ጸሃፊን በተመለከተው አድራሻ ማግኘት ይችላሉ (0118-962183)።

Appendix 6: Amharic version of consent form I

**ለወላጅ/አሳዳጊ የስምምነት መግለጫ ቅጽ ዕድሜያቸው 12-17 ዓመት**

**የጥናቱ ርእስ፡-** የቫይቫክስ ወባ መድሃኒቶች ፈዋሽነት ማረጋገጫ ጥናት

እኔ ወላጅ/አሳዳጊ, ልጄ በፈቃደኛነት በጥናት ተሳታፊ እንዲሆን የፈቀድኩት የተሳታፊነት ማሳወቂያ ቅፁን ከመፈረሜ አስቀድሞ ሥለጥናቱ አካሄድ እና ከዚህ ቀጥሎ የተዘረዘሩትን የጥናቱ ተሳታፊ መብቶች ግልፅ በሆነ መልኩ ከመረጃ ቅፁ አንብቤ ተረድቻለሁ /ተነቦልኝ በግልፅ ተብራርቶልኛል፡፡

**አግኝቻለሁ / አላገኘሁም፤** ስለ ጥናቱ ተገቢውን መግለጫ ማግኘቴን (ያክብቡ)

**ተረድቻለሁ / አልተረዳሁም፤** በተለያዩ ጊዜያት የልጄን በመብጣት እና ከክንዱ ላይ ስለሚወሰድ ናሙናና ስለጥናቱ ተጨባጭ ውጤት (ያክብቡ)

**ተስማምቻለሁ / አልተስማማሁም**፤ የተወሰነ ናሙና እስከ ጥናቱ መጨረሻ ጊዜ ድረስ እንዲቀመጥና ምርምር እንዲደረግበት (ያክብቡ)።

**ተስማምቻለሁ / አልተስማማሁም**፤ መጠይቁን ለማሟላት የቃል ጥያቄ ለማድረግ (ያክብቡ)

**ተስማምቻለሁ / አልተስማማሁም**፤ በጥናቱ ልጄ ለመሳተፉ (ያክብቡ)

መፃፍ ለማይችሉ የጣት እሻራ አዚህ ያሳርፉ

ወላጅ/ያሳዳጊ

ገለልተኛ ምስክር

የተሳታፊ ስም፦ ______________________________________

የተሳታፊ ፊርማ-------------------------------ቀን------------------

የተሳታፊ ቤተሰብ ስም፦ ______________________________________

የተሳታፊ ቤተሰብ ፊርማ፦ _______________ቀን፦________________

የገለልተኛ ምስክር ስም፦ _________________________________________________

የገለልተኛ ምስክር ፊርማ፦ ____________________ቀን________________

የተመራማሪው/ዋሰም፦_________________የተመራማሪው/ዋ ፊርማ፦_________ቀን፦__________
